# RGS5 augments astrocyte activation and facilitates neuroinflammation via TNF signaling

**DOI:** 10.1186/s12974-023-02884-w

**Published:** 2023-09-06

**Authors:** Shu Yin, Xin-yue Ma, Ying-feng Sun, Yan-qing Yin, Ying Long, Chun-lai Zhao, Jun-wei Ma, Sen Li, Yan Hu, Ming-tao Li, Gang Hu, Jia-wei Zhou

**Affiliations:** 1 Institute of Neuroscience, State Key Laboratory of Neuroscience, CAS Center for Excellence in Brain Science, Intelligence Technology, Chinese Academy of Sciences, 320 Yueyang Road, Shanghai, 200031 China; 2 Department of Pharmacology, Nanjing University of Chinese Medicine, Nanjing, 210023 Jiangsu China; 3 Center for Brain Disorders Research, Center of Parkinson’s Disease, Capital Medical University, Beijing Institute for Brain Disorders, Beijing, 100053 China; 4 Guangdong Provincial Key Laboratory of Brain Function, Disease, Department of Pharmacology, Zhongshan School of Medicine, Sun Yat-Sen University, Guangzhou, 510080 China; 5 School of Future Technology, University of Chinese Academy of Sciences, Beijing, 100049 China; 6 Shanghai Center for Brain Science, Brain-Inspired Intelligence Technology, Shanghai, 201210 China; 7 Co-Innovation Center of Neuroregeneration, School of Medicine, Nantong University, Nantong, 226001 Jiangsu China

**Keywords:** Astrocytes, Neuroinflammation, Parkinson’s disease, RGS5, TNFR

## Abstract

Astrocytes contribute to chronic neuroinflammation in a variety of neurodegenerative diseases, including Parkinson's disease (PD), the most common movement disorder. However, the precise role of astrocytes in neuroinflammation remains incompletely understood. Herein, we show that regulator of G-protein signaling 5 (RGS5) promotes neurodegenerative process through augmenting astrocytic tumor necrosis factor receptor (TNFR) signaling. We found that selective ablation of *Rgs5* in astrocytes caused an inhibition in the production of cytokines resulting in mitigated neuroinflammatory response and neuronal survival in animal models of PD, whereas overexpression of *Rgs5* had the opposite effects. Mechanistically, RGS5 switched astrocytes from neuroprotective to pro-inflammatory property via binding to the receptor TNFR2. RGS5 also augmented TNFR signaling-mediated pro-inflammatory response by interacting with the receptor TNFR1. Moreover, interrupting RGS5/TNFR interaction by either RGS5 aa 1–108 or small molecular compounds feshurin and butein, suppressed astrocytic cytokine production. We showed that the transcription of astrocytic *RGS5* was controlled by transcription factor early B cell factor 1 whose expression was reciprocally influenced by RGS5-modulated TNF signaling. Thus, our study indicates that beyond its traditional role in G-protein coupled receptor signaling, astrocytic RGS5 is a key modulator of TNF signaling circuit with resultant activation of astrocytes thereby contributing to chronic neuroinflammation. Blockade of the astrocytic RGS5/TNFR interaction is a potential therapeutic strategy for neuroinflammation-associated neurodegenerative diseases.

**Supplementary Information:**

The online version contains supplementary material available at 10.1186/s12974-023-02884-w.

## Introduction

Chronic neuroinflammation is associated with various neurodegenerative diseases such as Alzheimer’s disease (AD) and Parkinson's disease (PD). Activated microglia and astrocytes in the brain are major players in persistent neuroinflammation, which occurs well before neuronal loss, contributes to the onset and progression of neurodegenerative diseases [1, 2]. An increasing body of evidence suggests that astrocytes, which account for 50% of the cells in the human central nervous system (CNS), substantially contributes to neuroinflammation and the death of neurons in neurodegenerative diseases [3–6]. In the context of PD, activated astrocytes promote neuroinflammation and dopaminergic (DA) neuron degeneration through multiple mechanisms, including increased permeability of the blood–brain barrier and recruitment of leukocytes into the CNS via the secretion of chemokines [7–9]. A most recent study shows that reactive astrocytes release toxic factor long-chain saturated lipid which kills cells in the CNS [10], suggesting a key role of reactive astrocytes in neurodegeneration. Understanding the precise molecular basis of astrocytic inflammatory response is vital for the development of a novel therapeutical strategy for PD and other neurodegenerative diseases.

To date, tumor necrosis factor-α (TNF-α) has been considered as one of the most pivotal cytokines among a variety of inflammatory mediators in immune homeostasis. TNF-α is a pleiotropic cytokine regulating innate, adaptive systems through TNF receptor 1 (TNFR1) and TNF receptor 2 (TNFR2). In patients with PD, TNF-α protein in both the substantia nigra (SN) tissue and cerebrospinal fluid correlates well with disease progression [11]. Likewise, there is an elevation of TNF-α levels in most commonly used experimental models of PD in which DA neurons are highly sensitive to TNF-α [12]. TNF-α is secreted by microglia and astrocytes responding to classical inflammatory mediators [13, 14]. Combined treatment with TNF-α, interleukin (IL)-1α and complement component 1q (C1q) released by activated microglia can activate astrocytes, resulting in the conversion of astrocytes to neurotoxic A1 phenotype [9, 15, 16]. It is also recognized that in addition to PD, TNF-α also contributes a sustained astrogliosis in multiple neurological diseases, such as stress and traumatic brain injury [17, 18], suggesting that TNF-α-mediated astrogliosis is an integral player in the pathogenesis of a variety of neurological diseases. However, it remains to be investigated how TNF-α signals elicit such robust and long-lasting cellular responses.

Regulator of G protein signaling (RGS) proteins were originally identified as negative mediators of G-protein coupled receptors (GPCR) [19]. To date, there are at least 20 distinct RGS proteins which are classified into seven subfamilies, including RZ, R4, R7, R12, RA, GEF and GRK. Among them, the R4 subfamily, represents the least structurally and functionally complex of RGS family, that comprises the RGS domain, segments that are determinants of RGS interaction with G-proteins α subunits, and the structural basis underlying its GTPase-activating activity (GAP) [20–22]. Recent studies have implicated the involvement of RGS proteins in PD. For instance, RGS10 expressed in microglia, modulates key signaling pathways that are important for nigral DA neuron survival and/or proper motor control [23, 24]. RGS14 is among the genes that were downregulated in the both multiple system atrophy and PD putamen [25]. Given these points, we thus hypothesized that RGS proteins in glial cells may have functional contributions to chronic neuroinflammation and neurodegeneration.

In this study, we identify a RGS5-dependent mechanism for TNFR signaling as a crucial pathway in the regulation of neuroinflammatory response in astrocytes. Selective ablation of *Rgs5* in astrocytes causes a marked inhibition in TNF-α-induced inflammatory response and mitigated neuronal survival in animal models of PD. In contrast, selective overexpression of *Rgs5* in astrocytes causes an increase in the production of cytokines, and resulted in increased toxic protein aggregation and mortality in human α-synuclein A30P mutant transgenic (Tg) mice. RGS5 reverses the role of TNFR2 in neuroinflammation from anti-inflammatory to proinflammatory. RGS5 also enhances TNFR1 signaling leading to astrocytic inflammation. Thus, our study reveals the RGS5/TNFR axis as a critical determinant for astrocyte-driven immune response in PD pathogenesis.

## Methods

### Animals

All procedures involving the maintenance and handling were approved by the Institutional Animal Care and Use Committee and were in accordance with the US National Institutes of Health *Guide for the Care and Use of Laboratory Animals*. Adult or neonatal C57BL/6 mice were from Shanghai Laboratory Animal Center, Chinese Academy of Sciences. *Rgs5*-floxed mouse line was provided by G Hämmerling and B Arnold (DKFZ) [26]. Mouse glial fibrillary acidic protein promoter (mGFAP)-Cre transgenic mice (*B6.Cg-Tg(Gfap-cre)77.6Mvs/2J*) [27] and A30P-mutant human α-synuclein transgenic mice (Tg(THY1-SNCA*A30P)TS2Sud) [28] were purchased from the Jackson Laboratory (USA). Human GFAP (hGFAP)-Cre transgenic mice in a C57BL/6 genetic background, which were originally derived from *FVB-Tg(GFAP-Cre)25Mes/J* (Jackson Laboratory), were gifts from SM Duan. Mice harboring a TAM-inducible Cre recombinase transgene driven by the hGFAP promoter (*hGFAP-CreER*^*T2*^*)* in a C57BL/6 genetic background were provided by KD McCarthy [29]. *Ebf1*-floxed mice were created by the Shanghai Research Center for Model Organisms. Briefly, the floxed *Ebf1* allele was generated by introduction of loxP sites flanking the coding region of exon 3 of the *Ebf1* locus into the mouse genome. Recombinant embryonic stem cells were injected into C57BL/6 blastocysts to produce chimeras which were then crossed with C57BL/6 mice to produce mice heterozygous for the floxed-*Ebf1* allele (*Ebf1*^*flox/*+^). These animals were maintained on a 12-h light/dark cycle at 23 °C with food and water available ad libitum.

### Tamoxifen treatments

Tamoxifen (TAM, Sigma-Aldrich, T5648) was made freshly by dissolving in 95% corn oil (Sigma-Aldrich)/5% ethanol solution at room temperature with intermittent vortexing. Final concentration of TAM was 20 mg ml^−1^. Mice were injected intraperitoneally with 80–100 µg kg^−1^ daily for 5–8 consecutive days.

### Western blot analysis and quantification

Western blotting was performed as described previously [30]. The following primary antibodies were used: mouse anti-β-actin mAb (1:5000; Sigma-Aldrich, A5441); rabbit anti-IL-1β pAb (1:1000; Abcam, ab9722); rabbit anti-TNF-α pAb (1:1000; Abcam, ab9739); rabbit anti-GAPDH pAb (1:5000; Proteintech, 10494-1-AP); rabbit anti-alpha-synuclein pAb (1:2000; Cell Signaling, 2628); rabbit anti-TNFR2 pAb (1:1000; Beyotime, AF8199); rabbit anti-HA tag pAb (1: 1000; Abcam, ab9110); mouse anti-FLAG tag mAb (1: 5000; Sigma-Aldrich, F1804); The membrane was washed and incubated for 2 h at room temperature with the corresponding secondary antibodies: (a) HRP-conjugated goat anti-rabbit IgG (1:10,000; Jackson ImmunoResearch Laboratories, 115-035-003); (b) HRP-conjugated goat anti-mouse IgG (1:10,000; Jackson ImmunoResearch Laboratories, 115-035-003). Peroxidase activity was detected with SuperSignal WestPico chemiluminescent substrate (Pierce Biotechnology) and visualized and digitized with ImageQuant (LAS-4000, Fujifilm, Japan). Optical densities of bands were analyzed by using ImageReader software (Fujifilm, Japan). Protein levels, quantified by computer analysis as the ratio between each immunoreactive band and the levels of β-actin or GAPDH, were expressed as a percentage of vehicle-treated control.

### Immunofluorescence and confocal microscopy

Brain cryo-sections were incubated with one primary antibody followed by incubation with secondary antibody conjugated with either Alex488 or Alex555. For double-immunofluorescent staining, the same sections were then incubated with another primary antibody, followed by incubation with the appropriate secondary antibody and with Hoechst 33342 to show the nucleus. Sections were imaged using either a microscope (BX51, Olympus) equipped with a cooled CCD (DP72, Olympus) or a laser confocal microscope (Nikon A1, Japan). Data were obtained and processed using ImageJ (NIH, USA). In some cases, immunosignals were visualized by using 3,3-diaminobenzidine (Sigma-Aldrich). The following primary antibodies were used: mouse anti-GFAP mAb (1:500, Sigma-Aldrich, G3893); rabbit anti-GFAP pAb (1: 1,000; DAKO, Z0334); rabbit anti-Iba1 pAb (1:500; WAKO, 019-19741); rabbit anti-TH pAb (1:800; Chemicon, AB152); rabbit anti-GFP pAb (1:1000; Invitrogen, A11122); rabbit anti-TNFR1 pAb (1:200; Proteintech, 21574-1-AP); rabbit anti-TNFR2 pAb (1:200; Beyotime, AF8199); mouse anti-RGS5 pAb (1:500; Sigma-Aldrich, sc-390245).

### Cell counting

The number of tyrosine hydroxylase (TH)-positive cells was quantified in brain cryosections with typical morphology of the substantia nigra, as described previously [31]. Four series of cryosections were collected and every fourth section (25 μm) was used for quantification of TH^+^ neurons. The number of GFAP^+^ and Iba1^+^ cells was quantified using a similar approach. The assay was randomized and blind to the experimenter.

### Quantification of immunoreactivity

GFAP or Iba1-immunopositivities in the substantia nigra pars compacta (SNc) and the substantia nigra pars reticulata (SNr) and the integrated density of RGS5-immunopositive regions were estimated using ImageJ (NIH, USA) software, in 20 × images taken from 4–5 sections. Values are reported as average percentages of area ± SEM. The assay was randomized and blind to the experimenter.

### Primary astrocytic cultures

Glial cultures were prepared from Sprague-Dawley rat pups or *Ebf1*-deficient and wild-type C57BL/6 mouse pups at P0–P2, as previously described [32]. Briefly, the neonatal brains with hippocampus removed were dissociated and cells were plated at density of 5 × 10^7^ cells/75 cm^2^ flask (Corning, USA) in Dulbecco's modified Eagle's medium (DMEM) containing 10% fetal bovine serum (FBS). Culture media were switched to complete medium 24 h after plating and subsequently changed twice a week. Cultures were shaken vigorously to remove the top cell layer sitting over the astroglial monolayer to yield mainly type-I astrocytes. Astrocytic cultures were passaged once. The purity of primary astrocyte cultures after purification was more than 93% and cells were allowed to reach 90% confluence. Astrocytes were exposed to lipopolysaccharide (LPS, 500 ng/ml) or TNF-α (100 ng/ml).

### Enzyme linked immunosorbent assay (ELISA)

ELISA was performed using the Mouse TNF-α ELISA kit (Sangon Biotech; D721026) according to the manufacturer's instruction. In brief, primary astrocyte cultures were incubated with TNF-α (100 ng/ml) for 1 h. The cultures were washed with DMEM for 3 times before the cultured medium was switched to DMEM containing 10% FBS for 4 h. The medium was collected and centrifuged. Standard and samples were added to the microplate that have been pre-coated with anti-mouse TNF-α antibody. After incubation, biotin-conjugated anti-mouse TNF-α antibody was added. It was then combined with HRP-conjugated streptavidin to form an immune complex, then incubated and washed to remove unbound enzyme, and then added to the chromogenic substrate TMB to produce a blue color, and converted to the final yellow under the action of acid. Finally, the absorbance (OD) value was measured at 450 nm. The concentration of mouse TNF-α in the sample was proportional to the OD value. The concentration of mouse TNF-α in the sample can be calculated from a standard curve.

### Purification of glial cells from mouse brain by using MACS

Purification of glial cells by magnetic-activated cell sorting (MACS) was performed as described previously [33]. Briefly, adult mouse brains (2–5 months old) with the hippocampus removed were dissociated using Neural Tissue Dissociation Kit (T) (Miltenyi Biotec; 130-093-231) followed by washing with MACS buffer (sterile-filtered 0.5% BSA, 2 mM EDTA in PBS). Then GLAST^+^ cells were positively selected with anti-GLAST (ACSA-1) microbeads (Miltenyi Biotec; 130-095-826).

### Transcription factor luciferase reporter assay

Fragments encompassing the 5’-flanking region of the human RGS5 gene (2.4 kb; GenBank NT_004487) cloned into pGL-3 vector, were a gift from J Li (Harvard Medical School, USA) [34]. To carry out the promoter activity assay, HEK293T cells were co-transfected with the construct of Rgs5 promoter and an expression vector encoding Ebf1 cDNA (EF1α-Ebf1). The pGL3 vector or empty vector (EF1α) served as control. The cells were harvested for dual luciferase assay 36 h after transfection. Luciferase activity was assessed using the Dual-Luciferase assay system (Promega).

### Chromatin immunoprecipitation (ChIP)

ChIP was performed using the EZ-ChIP kit (Millipore; 17-371) according to the manufacturer's instruction. In brief, chromatin was isolated from mouse neuroblastoma N2a cells stably overexpressing Ebf1-tagged with HA or empty vector either in basal condition or challenged with LPS (1 µg/ml, 2 h). Monoclonal anti-HA antibody produced in mouse (Sigma-Aldrich, H3663) and anti-RNA Pol II antibody were used for IP. The promoter sequences corresponding to the upstream of the start site of Rgs5 were used for analysis. Primer sets corresponding to the regions are as follows: − 2128 to − 2057 bp from TSS, forward 5′-TCACATGAACTCCTTTGGGACA-3′, reverse 5′-ATGGAGGGTGATGTCTTGGC-3′; − 1678 to − 1580 bp from TSS, forward 5′-TAGCTAGGCATGGTAGCAAG-3′, reverse 5′-ATAGCTCACTGCATCATCAA-3′; − 1973 to − 1828 bp from TSS, forward 5′-TGTCCCAAGGAGTCTCTTCT-3′, reverse 5′-ACAAACTGTCCAAGTTCCGC-3′.

### Coimmunoprecipitation analysis (Co-IP)

Co-IP was performed as described previously [35]. Approximately 3.5 × 10^6^ HEK293T cells were harvested ∼24 h after transfection and lysed in 450 µl of cell lysis buffer for Western and IP (Beyotime; P0013). Lysates were precleared by incubating with protein A/G-PLUS agarose beads (Santa Cruz, sc-2003) for 1 h. And the primary antibodies, anti-HA tag antibody (Sigma-Aldrich, H3663) and anti-FLAG tag antibody (Sigma-Aldrich, F1804) were used.

### Plasmid constructions and virus package

The full-length coding region of human RGS5 isoform 1, TNFR1, TNFR2 and their truncations were isolated from 293T cell cDNAs by PCR amplification and cloned into mammalian expression lentivirus vectors (LV) or adeno-associated virus (AAV) vectors. HA-TNFR2 construct was purchased from the Sino Biological (HG10417-NY). The cloned genes were under control of EF1α or CMV or GFAP promoter. All constructs were verified by sequencing. Lentivirus or AAV were packaged by OBiO Technology (Shanghai) Corp. The GFAP promoter was provided by Dr. Michael Brenner through the Alabama Neuroscience Blueprint Core.

### Stereotaxic injection of lentivirus or AAV, LPS or 6-OHDA

Lentivirus or AAV or LPS (5–8 µg, Sigma-Aldrich) or 6-OHDA (4 µg, Sigma-Aldrich) were directly injected into the adult mouse cerebral lateral ventricles or SN or striatum using the following coordinates: cerebral lateral ventricles, AP, − 1 mm, ML, − 1.5 mm, DV, − 2.3 mm; SN, AP, − 2.85 mm, ML, − 1.3 mm, DV, − 4.6 mm; striatum, AP, + 0.5, ML, − 1.85 mm, DV, − 3.6 mm from the bregma. Three weeks after LPS injection or 2 weeks after 6-OHDA injection, mice were killed and perfused with 4% paraformaldehyde in 0.1 M PB (pH 7.4) and coronal cryo-sections at a thickness of 25 µm were prepared for immunohistochemistry.

### Intraperitoneal injection of LPS

Adult mice were given a single intraperitoneal injection of 5 mg/kg LPS (Sigma-Aldrich) or vehicle (saline). 24 h post-injection, the animals were killed by rapid decapitation, and the striatum and VM were dissected and processed for Western blot or qPCR analysis. In some cases, mice received intraperitoneal LPS injections (2.5 mg/kg/day for 2 consecutive days).

### RNA isolation and quantitative PCR

Isolation of total RNA was performed as described previously [36]. Briefly, brain tissue was homogenized in TRIzol reagent (Invitrogen, Carlsbad, CA, USA). cDNA was synthesized from 1 µg of extracted total RNA using M-MLV Reverse Transcriptase kit (Invitrogen) according to the manufacturer’s protocol. Quantitative PCR was performed with SYBR-Green premix Ex Taq (Takara, Japan) and detected by a Real Time PCR System (Roche LightCycler 480 or Rotorgene 6000). β-Actin was used as an internal control gene. qPCR primers were designed using Primer Picking Program. Following PCR amplification, a first derivative melting-curve analysis was performed to confirm the specificity of the PCR. The relative fold difference in mRNA between samples was calculated by comparing the threshold cycle (*C*t) at which product initially appeared above background according to: 2^− (∆*C*t)^, where *∆C*t is the difference between control group and a treatment group.

### NanoBRET/NanoBit protein:protein interaction assays

The assay was performed using NanoBRET™ PPI Starter System (Promega, N1811) according to the manufacturer instructions. In brief, 2 × 10^5^ cells were plated into a 24-well plate (Corning) and maintained for 24 h at 37 °C, 5% CO_2_. Then cells were transfected with a mixture of 0.5 µg fusion vector containing HaloTag and 0.05 µg fusion vector containing NanoLuc by using PEI (Thermo Fisher Scientific, USA). The NanoBRET™ assay was optimized firstly to confirm the positive of target protein in vectors. Twenty-four hours later, the cells were plated into a 384-well microplate (Corning, 3765). The media was switched to Opti-MEM (Gibco, 11058021) containing 4% FBS with or without 100 nM HaloTag® NanoBRET™ 618 Ligand (Promega, N1662) per well. Plates were incubated overnight followed by the addition of TNF-α (final concentration, 20 or 100 ng/ml) or vehicle per well. And after 1 h, 5 × NanoBRET™ Nano-Glo® Substrate (Promega, N1662) in Opti-MEM was added to the cultures for taking measurements. Donor emission (460 nm) and acceptor emission (618 nm) were measured within 10 min following addition of substrate using Envision (PerkinElmer, USA). We calculated BRET ratio (mBU) = BU618 ligand-BUno ligand (BU = 618 nm_Em_/460 nm_Em_).

For NanoBit assay, cells were co-transfected with vector expressing the SmBit Nano luciferase fused to the amino terminus of RGS5 and the LgBit Nano luciferase fused to the carboxy terminus of TNFR1 or TNFR2. Transfected cells were then seeded onto a white, clear-bottom 384-well plate with Opti-MEM medium containing 4% FBS. Cells were incubated with compounds for 6 h before addition of the luciferase substrate.

### RNA-seq analysis

The GLAST^+^ cells were purified from whole brain tissue of 3-month-old *Rgs5*^*hGFAP−creER*^ and littermate control mice following administration of saline or LPS (2. 5 mg/kg/day for 2 consecutive days) The brain tissues were homogenized in TRIzol reagent (Invitrogen, Carlsbad, California, USA). A total amount of 600 ng RNA per sample was used as input material for the RNA sample preparations. Sequencing services were provided by the Novogene Technology Co., Ltd. Sequencing libraries were generated using NEB Next® Ultra RNA Library Prep Kit for Illumina® (NEB, USA) following manufacturer’s recommendations and index codes were added to attribute sequences to each sample. Briefly, mRNA was purified from total RNA using poly-T oligo-attached magnetic beads. Fragmentation was carried out using divalent cations under elevated temperature in NEBNext First Strand Synthesis Reaction Buffer (5X). First strand cDNA was synthesized using random hexamer primer and M-MuLV Leading Edge Genomic Services & Solutions Reverse Transcriptase (RNase H). Second strand cDNA synthesis was subsequently performed using DNA polymerase I and RNase H. Remaining overhangs were converted into blunt ends via exonuclease/polymerase activities. After adenylation of 3′ ends of DNA fragments, NEB Next Adaptor with hairpin loop structure were ligated to prepare for hybridization. In order to select cDNA fragments of preferentially 250–300 bp in length, the library fragments were purified with AMPure XP system (Beckman Coulter, Beverly, USA). Then 3 µl USER Enzyme (NEB, USA) was used with size-selected, adaptor-ligated cDNA at 37 °C for 15 min followed by 5 min at 95 °C before PCR. Then PCR was performed with Phusion High-Fidelity DNA polymerase, Universal PCR primers and Index (X) Primer. At last, PCR products were purified (AMPure XP system) and library quality was assessed on the Agilent Bioanalyzer 2100 system [37].

#### Differential expression analysis

Reference genome and gene model annotation files were downloaded from genome website directly. Index of the reference genome was built using Hisat2 v2.0.5 and paired-end clean reads were aligned to the reference genome using Hisat2 v2.0.5. Feature Counts v1.5.0-p3 was used to count the reads numbers mapped to each gene. And then FPKM of each gene was calculated based on the length of the gene and reads count mapped to this gene. Differential expression analysis of two conditions was performed using the edgeR R package (3.18.1). The *P* values were adjusted using the Benjamini and Hochberg method. Corrected *P*-value of 0.05 and absolute fold change of 2 were set as the threshold for significantly differential expression.

#### GO and KEGG enrichment analysis of differentially expressed genes

Gene Ontology (GO) enrichment analysis of differentially expressed genes was implemented by the cluster Profiler R package, in which gene length bias was corrected. GO terms with corrected *P* value less than 0.05 were considered significantly enriched by differential expressed genes. KEGG is a database resource for understanding high-level functions and utilities of the biological system, such as the cell, the organism and the ecosystem, from molecular-level information, especially large-scale molecular datasets generated by genome sequencing and other high-through put experimental technologies (http://www.genome.jp/kegg/). We used cluster Profiler R package to test the statistical enrichment of differential expression genes in KEGG pathways.

### Establishment of HEK293T cells stably expressing NF-κB luciferase reporter gene

Human embryonic kidney cell line 293T cells (HEK293T) cells were co-infected with pLenti-NF-κB-GFP-luciferase reporter (NF-κB-Luc-blasticidin (V3), Ji Manchu Biotechnology (Shanghai Co., Ltd, China) and EBF1 or RGS5 gene-expressing lentivirus. Cells co-infected with pLenti-NF-κB-GFP-luciferase reporter and GFP-expressing lentivirus (pLenti-FLAG-2A-GFP) served as negative control. The co-infected clones were isolated and expanded by using flow cytometry. Luciferase activity was measured using Ensight (PerkinElmer, USA).

### Behavior assays

All behavior assays were randomized and blind to the experimenter. And all behavior tests were mostly begun at 2:00 pm. Animals were acclimatized to the testing room for 30–60 min prior to the test. Each animal was put back to its home cage after each trial. Besides, the test materials were cleaned with 75% ethyl alcohol and permitted to dry between every test.

#### Pole test

The method was modified from the reported protocol [38, 39]. Briefly, a vertical wooden rough-surfaced pole (1 cm in diameter, 50 cm in height) was selected and the base of the pole was placed in a cage. An animal was placed head-downward on the top of the pole, recorded the time until the animal reached the floor with its fore paws. Each animal was tested six times, at an interval of 10 min. And the average value was used as the final results. If the animal fell or slipped down during the descent, the actual time for animal to reach the floor was taken into account. And if the animal stopped during the descent, the result was invalid.

#### Open field test

The open field test was used with a 40 × 40 × 40 cm (width × length × height) square box. Each individual animal was placed in the center field at the start of the test [35]. Following 30 s of adaptation, spontaneous exploration was recorded for 30 min. And the total distance traveled was analyzed by a video tracking software package (EthoVision XT).

#### Wire hang test

An animal was placed on a wire lid of the cage, and made sure the animal gripped the lid with its four paws while inverting the lid [39, 40]. The vertical distance between the wire lid and the bottom of the cage was 40 cm. Each animal was tested three trials. The time taken for animal to fall from the cage lid to the bottom of the cage was recorded and the average value was calculated as the final result. If the animal did not fall after 60 s, 60 s was recorded as the test result.

### Statistical analysis

Statistical analysis was performed using GraphPad software (GraphPad Prism v8.0; GraphPad Software). Data presented as mean ± SEM were submitted to two-side *t*-test or one-/two-way ANOVA followed by Dunnett’s test. Differences were considered significant at values of *P* < 0.05.

## Results

### Astrocytic RGS5 is required for inflammation-associated neurodegeneration

**Fig. 1:**
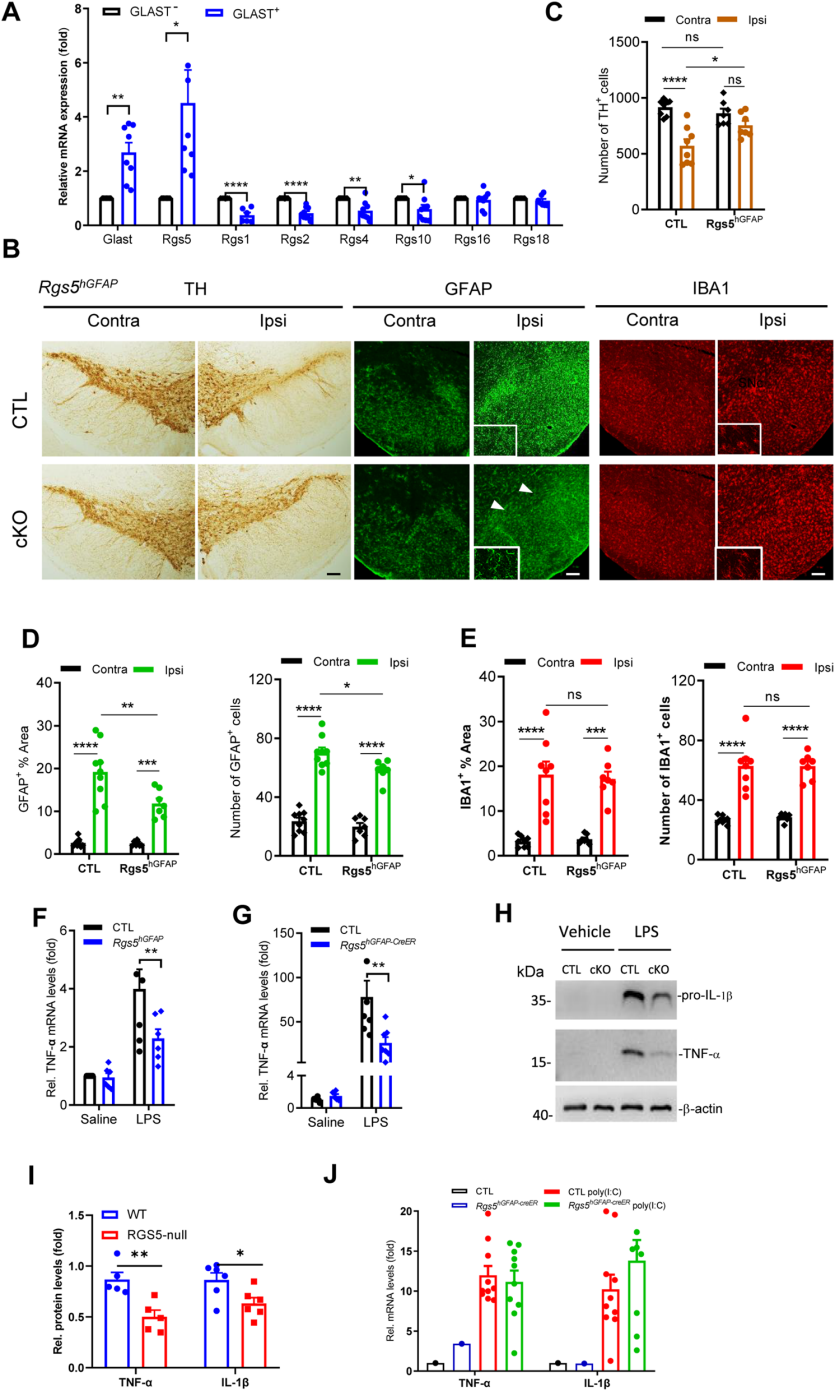
Preferential ablation of *Rgs5* in astrocytes attenuates inflammatory responses and DA neurodegeneration in LPS-challenged mice. **A** Representative graph showing relative mRNA levels of R4 and non-R4 subfamily members in GLAST^+^ astrocytes isolated from the whole brain of 2-month-old mice using the MACS. *n* = 7–9 per group, paired *t*-test. **B** Immunohistochemical staining for TH, GFAP and IBA1 on the ventral mesencephalon of adult *Rgs5*^*hGFAP*^ cKO and their littermate controls received a single intra-nigral injection of LPS. The inserts are enlarged views of the regions of the corresponding photos. Scale bars, 100 μm. **C** Quantitative data of number of TH^+^ cells in B. *n* = 7. **D** Quantification of immunoreactivity or immunoreactive cells for GFAP shown in **B**. *n* = 7–9. **E** Quantification of immunoreactivity or immunoreactive cells for IBA1 shown in **B**. *n* = 7–8. **F** Representative graph showing relative TNF-α mRNA levels in the brain of WT and *Rgs5*^*hGFAP*^ cKO mice treated with either saline or LPS (5 mg/kg, 4 h). *n* = 5–7 per group. **G** Reduction of pro-inflammatory mediators in the substantia nigra of *Rgs5*^*hGFAP−CreER*^ mice treated with either saline or LPS (2.5 mg/kg, daily for 2 consecutive days, *n* = 6–11). **H** Representative Western blot analysis of pro-IL-1β and TNF-α in the astrocytes following LPS challenge (500 ng/ml, 5 h). **I** Quantification of **H**, *n* = 5–6. **J** Ablation of *Rgs5* in astrocytes does not alleviate the poly(I:C)-induced inflammation in the SN. poly(I:C) HMW, 2.5 mg/kg, daily for 2 consecutive days. Data are expressed as mean ± SEM. Two-way ANOVA followed with Bonferroni’s multiple comparisons test or Student’s *t* test. *P < 0.05; **P < 0.01; ***P < 0.001; ****P < 0.0001

We first analyzed RGS expression in astrocytes, focusing on the R4 subfamily which has the least structurally and functionally complex of RGS family [19]. Glutamate aspartate transporter (GLAST)^+^ astrocytes were isolated from adult mouse brain, by using the magnetic activated cell sorting (MACS) technique. qPCR analysis revealed that relative mRNA expression levels of *Rgs5*, but not other R4 subfamily members, including *Rgs1, Rgs2, Rgs4, Rgs16* and *Rgs18,* in these astrocytes were higher than that in GLAST^–^ cells (Fig. 1A). And the levels of *Rgs10*, a non-R4 subfamily member which plays a role in microglia [23, 24], were significantly decreased in GLAST^+^ astrocytes. Double immunofluorescent staining revealed co-localization of RGS5 and glial fibrillary acid protein (GFAP), the main intermediate filament in astrocytes, in the SN of adult wild-type mice (Additional file 1: Fig. S1A). In A30P-mutant α-synuclein transgenic (Tg) mouse line which is a widely used genetic PD animal model [28, 41], a pronounced increase in RGS5 immunosignals was observed in GFAP^+^ astrocytes in the transgene compared to wild-type mice (Additional file 1: Fig. S1B, C). These analyses indicate that Rgs5 is expressed in astrocytes and upregulation of RGS5 is associated with neurodegeneration.

To unravel the role of astrocytic *Rgs5* in neuroinflammatory response, we bred *Rgs5*^*flox/flox*^ mice with either human GFAP *(hGFAP)-Cre* or *hGFAP-CreER* transgene to generate *Rgs5*^*hGFAP*^ cKO or *Rgs5*^*hGFAP−CreER*^ cKO mice, respectively. Pronounced decreases in *Rgs5* mRNA expression were confirmed in multiple brain regions of *Rgs5* cKO mice (Additional file 1: Fig. S1D). Previous studies have confirmed that a single intra-mesencephalic injection of LPS, an activator of microglial TLR4 signaling in the CNS, is sufficient to induce the loss of DA neurons in rodents, mimicking some features of PD pathology [42–45]. Using this animal model, we assessed the impact of *Rgs5* deficiency on the response of astrocytes and TH^+^ neuron loss. We found that 3 weeks after surgery, selective ablation of *Rgs5* in astrocytes significantly attenuated astrogliosis in the SN ipsilateral to the lesion compared with those in littermate controls, as manifested by reduced immunoreactivities of the astrocyte specific marker, GFAP (Fig. 1B, D). As a result, LPS-induced DA neuron loss was ameliorated in the same brain region (Fig. 1B, C). In contrast, the activation of ionized calcium binding adaptor molecule 1 (IBA1)-positive microglia was comparable in both groups induced by LPS (Fig. 1B, E).

To validate the results shown in Fig. 1B, C, a second mouse line lacking *Rgs5* in astrocytes, *Rgs5*^*flox/flox*^*;*mouse *GFAP-Cre (Rgs5*^*mGFAP*^) cKO mice [27], was used. Similarly, a protective effect on DA neurons was also observed on the lesion side of *Rgs5*^*mGFAP*^ mice following a single intra-mesencephalic injection of LPS compared with wild-type animals (Additional file 1: Fig. S1E, F). Together, these data suggest that RGS5 is necessary for LPS-induced astrocyte activation and DA neuron degeneration.

We found that the attenuation of DA neuron loss observed in LPS-challenged *Rgs5* cKO mice was associated with the downregulation of proinflammatory mediators. There was a marked reduction in levels of *Tnf*α in *Rgs5*^*hGFAP*^cKO mice following 4 h exposure to LPS compared with their littermate control (Fig. 1F). These results were confirmed in experiments with third mouse line lacking *Rgs5* in astrocytes (*Rgs5*^*hGFAP−creER*^) (Additional file 1: Fig. S1G) in an alternative inflammation-based animal model, which had extended exposure to LPS (2.5 mg/kg, daily for 2 consecutive days). These mice displayed a more pronounced reduction (> 50%) in mRNA levels of *Tnf*α as compared to wild-type littermate control administered with LPS (Fig. 1G), suggesting that RGS5 is a key modulator in astrogliosis. In support of this, such suppressive effect was observed in primary cultured astrocytes with knockdown of *Rgs5* (Fig. 1H, I and Additional file 1: Fig. S1H). Moreover, the neuronal protective effects of selective ablation of *Rgs5* in astrocytes can be extended to other PD animal models. In 6-hydroxydopamine (6-OHDA)-induced mouse model of PD, we found that selective ablation of *Rgs5* in astrocytes robustly attenuated DA neuron loss and activation of astrocytes and microglia (Additional file 1: Fig. S2). Furthermore, astrocyte-specific deficiency of *Rgs5* failed to hinder the neuroinflammation induced by poly(I:C), a ligand for Toll-like receptor (TLR)-3 (Fig. 1J), indicating a tendency of the RGS5-associated mechanism to favor the TLR-4-mediated inflammatory response. Together, these data support an idea that RGS5 plays a key role in astrocyte-mediated neuroinflammation in animal models of PD.

### Astrocytic RGS5 enhances inflammatory response and neurodegeneration

**Fig. 2:**
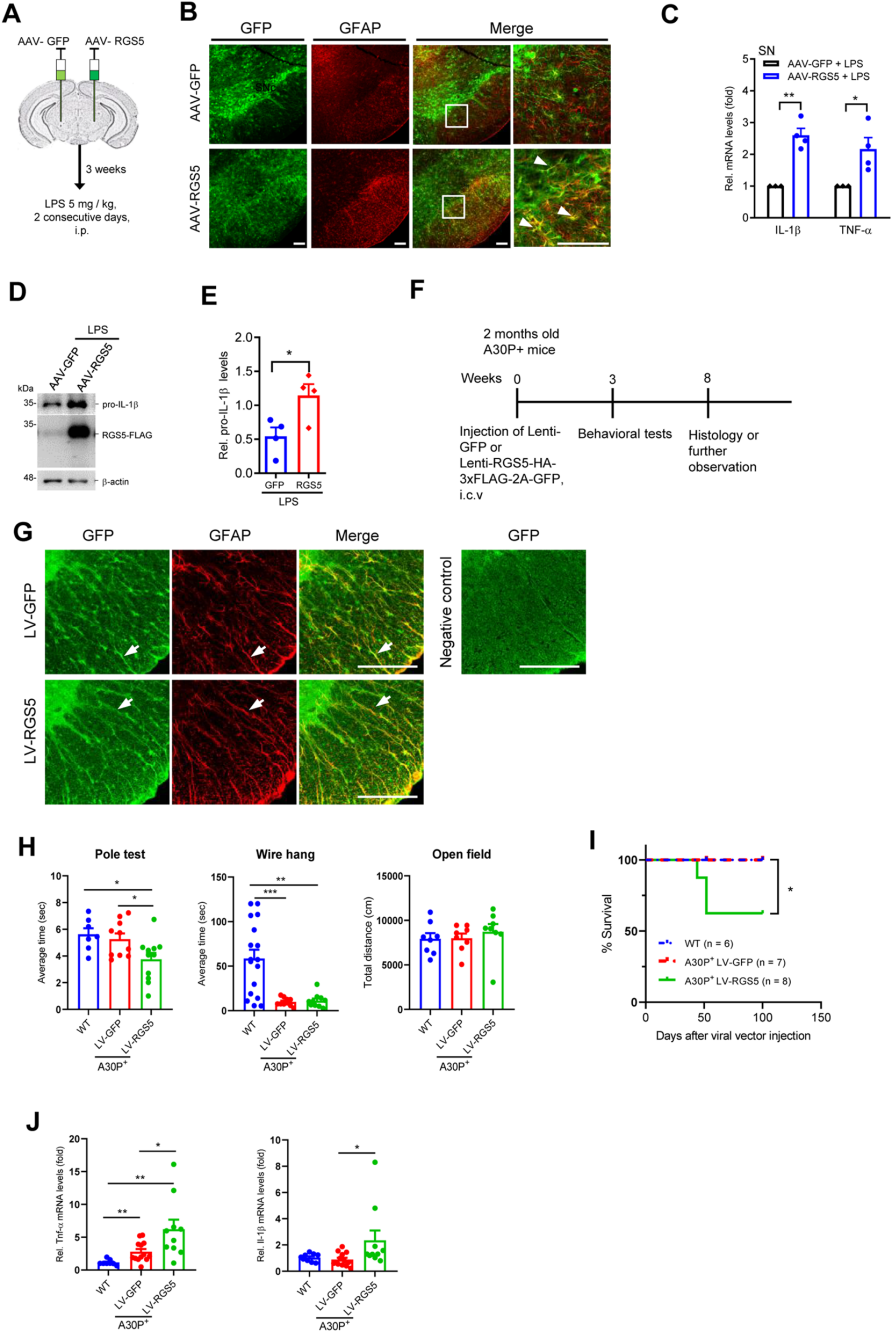
Selective *Rgs5* overexpression in astrocytes induces expression of pro-inflammatory mediators and promotes α-synuclein aggregation. **A** Schematic drawing showing the stereotaxic injection of AAV2/5 virus into mouse substantia nigra. Mice received unilateral viral injection of AAV-RGS5-FLAG-2A-GFP driven by GFAP promoter (AAV-RGS5) on one side and AAV-GFP on the other side, and were harvested 3 weeks post-injection. **B** Immunofluorescence staining for GFAP on the SN of mice injected with AAV-GFAP-RGS5 or AAV-GFAP-GFP. The right panel is an enlarged view of the rectangular area of the corresponding photo. Arrows indicate double-labeled cells. Scale bars, 100 μm. **C**–**E** Effect of *Rgs5* overexpression on the production of pro-inflammatory mediators in the SN upon LPS stimulation as revealed by qPCR (**C**) or Western blot analysis (**D** and **E**). LPS, 2.5 mg/kg/day, i.p., 2 consecutive days, *n* = 4 per group. **F** Diagram indicating the experimental design of lentivirus-mediated Rgs5 overexpression targeting astrocytes in A30P-mutant α-synuclein transgenic mice. Lenti-GFP (LV-GFP) or Lenti-RGS5-HA-3xFLAG-2A-GFP (LV-RGS5) driven by GFAP promoter were stereotaxically injected into the lateral ventricle of the brain. **G** Immunofluorescence staining for GFAP on the spinal cord of A30P-mutant a-synuclein transgenic mice injected with LV-RGS5 or LV-GFP. Arrows indicate double-labeled cells. Scale bars, 100 μm. **H** Behavioral changes of A30P-mutant α-synuclein transgenic mice injected with LV-RGS5 or LV-GFP, as assessed by the pole test, wire hang test and open field test. *n* = 8–11. **I** Kaplan–Meier survival rates in wild-type mice and A30P mutant α-synuclein transgenic mice receiving LV-RGS5, LV-GFP, respectively. Log-rank test, *n* = 6–8 per group. **J** Increased TNF-α and/or IL-1β transcription in RGS5-overexpressing A30P-mutant a-synuclein transgenic mouse spinal cord as revealed by qPCR analysis. *n* = 8–13, two-side *t* test. Data are expressed as mean ± SEM. *P < 0.05; **P < 0.01; ***P < 0.001; ****P < 0.0001

Next, we investigated whether RGS5 is capable of promoting inflammation. To this end, wild-type mice were subjected to unilateral intra-mesencephalic injection of AAV-GFAP-RGS5 (2 × 10^12^ TU/ml; 1 µl) driven by the astrocyte-specific GFAP promoter followed by a single intraperitoneal administration of LPS (Fig. 2A). As expected, overexpression of RGS5 selectively in astrocytes caused remarkable increases in levels of proinflammatory mediators, including *Tnf*α and *Il1*β in mice (Fig. 2B–E). In accordance with these changes observed in vivo, similar results were observed in primary cultured astrocytes (Additional file 1: Fig. S3A, B), suggesting that the RGS5-mediated inflammatory response occurs in a cell-autonomous fashion. NF-κB luciferase reporter activity assay showed that RGS5 may augment this process by activating NF-κB pathway, because there was a dramatic increase in NF-κB activity following overexpression of RGS5 (Additional file 1: Fig. S3C).

To further examine whether RGS5-mediated inflammation contribute to neurodegeneration, we used A30P-mutant α-synuclein transgenic (Tg) mouse line, a widely used genetic animal model of PD, which develops a neurodegenerative syndrome that manifests in the spinal cord rather in the brain [28, 41]. To achieve a higher transfection efficacy in astrocytes in the spinal cord as reported [46], the Tg mice were subjected to the injection of Lenti-GFAP-RGS5 (2 × 10^8^ TU/ml; 20 µl) in the lateral ventricle (Fig. 2F). We found that following selective overexpression of RGS5 in astrocytes (Fig. 2G), A30P-mutant α-synuclein Tg mice exhibited a rapid decline in motor function with the hind limbs most affected, as evidenced by a prominent reduction in grip strength with abnormal hind limb clasping in the pole test as compared with control (Fig. 2H), the behavioral phenotypes manifested in early stages of neurodegenerative disease [41, 47]. Notably, A30P-mutant α-synuclein Tg mice co-expressing RGS5 showed increased mortality with increasing age compared to control (Fig. 2I). Consistently, the transcription levels of proinflammatory mediators were increased in A30P-mutant α-synuclein transgenic mouse spinal cord overexpressing RGS5 (Fig. 2J). Taken together, these data suggest that astrocytic RGS5 plays a key role in neuroinflammation and degeneration in a mouse model of PD.

### Astrocytic RGS5 controls the expression of inflammation-associated genes

**Fig. 3:**
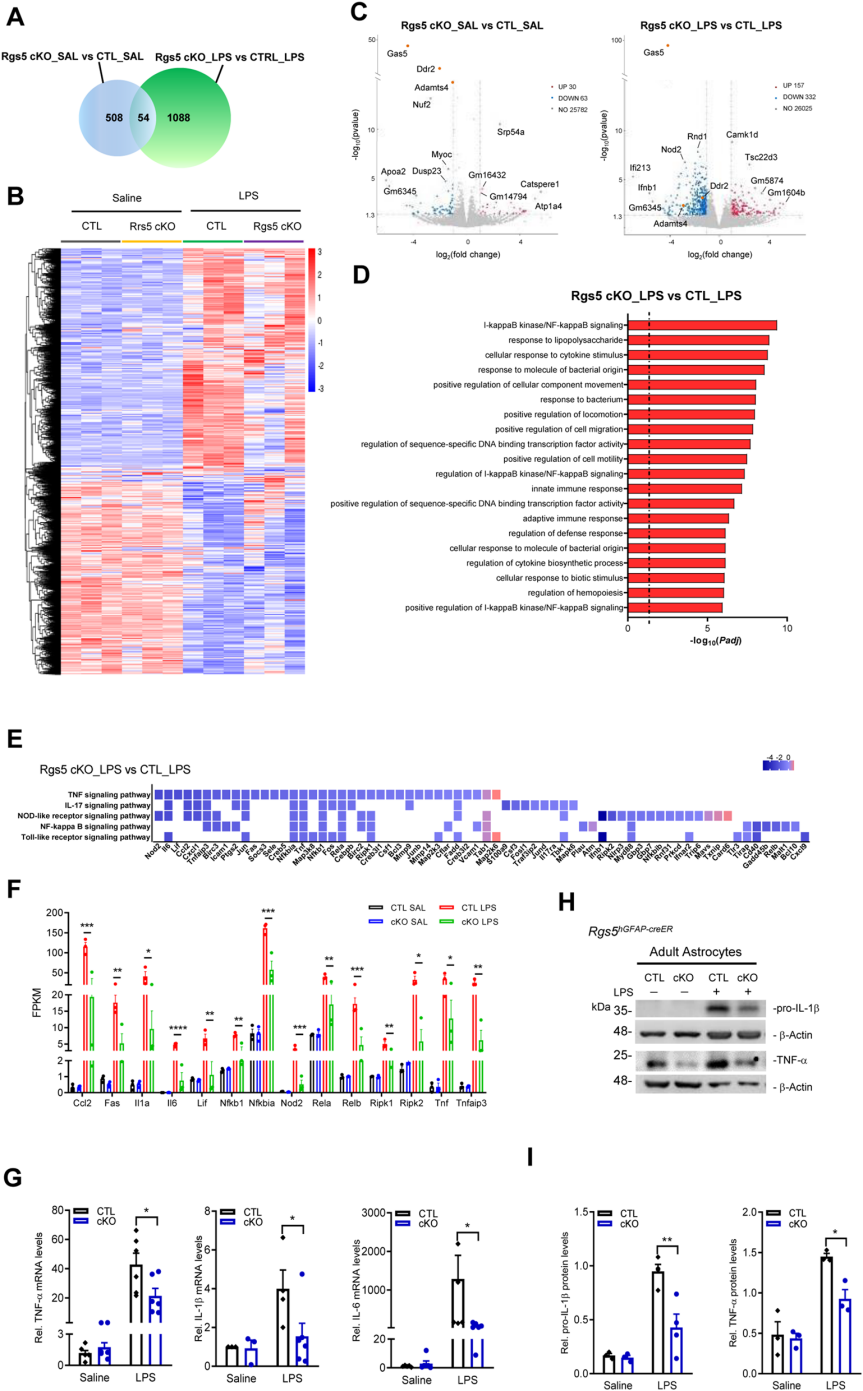
Downstream effectors of RGS5 contribute to inflammatory response. **A** A Venn diagram analysis of the differentially expressed genes (with ≥ twofold change and *P* < 0.05) in GLAST^+^ astrocytes between *Rgs5*^*hGFAP−CreER*^ cKO or littermate control administered with saline or LPS. The sum of numbers shown in each circle is the total number of differentially expressed genes in the comparison groups. The overlapping part between the two circles is the number of differentially expressed genes that are common among the comparison groups. **B** Hierarchical clustering of up- or down-regulated genes (FPKM: fragments per kilobase of exon per million mapped reads) of GLAST^+^ astrocytes isolated from adult *Rgs5* cKO mice administered with LPS or saline. Profiles are shown in a color scale where blue is low and red is high. *n* = 3 per group. **C** Volcano plots of *P* values as a function of weighted fold change between *Rgs5* cKO and control groups with or without LPS. Red and blue dots represent the upregulated and downregulated mRNAs. Some genes with higher significance values are annotated. **D** The top 20 differentially regulated Gene Ontology (GO) terms by the combination of RGS5 ablation and LPS treatment. **E** A heatmap showing the differentially expressed genes annotated in immunological function-associated pathways in KEGG database in *Rgs5* cKO astrocyte compared to control groups. These indicated pathways are among the top 6 down-regulated pathways. **F** FPKM values of genes associated with the TNF signaling pathway and NF-kappa B signaling pathway. **G** Relative TNF-α and IL-6 mRNA levels in GLAST^+^ astrocytes isolated from the whole brain of *Rgs5*^*hGFAP−CreER*^ cKO and control animals treated with either saline or LPS using MACS. *n* = 6 per group. **H**, **I** Representative Western blots (**H**) showing expression of pro-IL-1β and TNF-α in GLAST^+^ astrocytes from *Rgs5*^*hGFAP−CreER*^ mice. **I** Quantitative data shown in **H**. *n* = 3. Data are expressed as mean ± SEM. Two-way ANOVA followed with Bonferroni’s multiple comparisons test or Student’s *t*-test. *P < 0.05; **P < 0.01; ***P < 0.001; ****P < 0.0001

To gain more insight into the molecular process regulated by RGS5, RNA-sequencing was carried out in GLAST^+^ astrocytes isolated from *Rgs5*^*hGFAP−CreER*^ cKO mice and littermate controls challenged with or without LPS. It was revealed that there were 508 differentially expressed genes (with > twofold change, *P* < 0.05) between *Rgs5* cKO mice and control mice under basal condition. Expression of dual specificity phosphatase 23 (DSP23), a structural gene found in astrocytes [48], was significantly downregulated in *Rgs5*-deficient astrocytes, indicating a perturbation of the gene network in these astrocytes. Following LPS administration, there were 1088 differentially expressed genes between *Rgs5* cKO LPS mice and control LPS (Fig. 3A). Hierarchical clustering analysis demonstrated a clear segregation of these differentially expressed genes in mice between genotypes (Fig. 3B). The expression of nucleotide-oligomerization domain receptor 2 (*Nod2*) and interferon-β1 (*Ifnb1*), two important regulators in the immune system, was significantly decreased in *Rgs5*-null astrocytes following LPS challenge, indicative of the suppression of inflammatory response in astrocytes deficient in *Rgs5*. Notably, there was an increase in mRNA levels of TSC22 domain family member 3 (TSC22D3), which is known to function as a transcriptional regulator with anti-inflammatory activity [49], in astrocytes of *Rgs5* cKO mice compared to control (Fig. 3C). These results suggest that RGS5 confers proinflammatory effects on the regulatory gene network associated with immune homeostasis.

Moreover, Gene Ontology (GO) enrichment analysis showed that *Rgs5*-null astrocytes were much less inflamed following LPS challenge, as the top 20 biological pathways most significantly enriched among the genes that differed were related to infection, inflammation and immunological response (Fig. 3D). Further comparison of the differentially expressed genes on Kyoto Encyclopedia of Genes and Genomes (KEGG) pathways in the mice with different genotypes with LPS challenge showed that TNF signaling, IL-17 signaling, NOD-like receptor signaling, NF-κB signaling and Toll-like receptor signaling were significantly suppressed following *Rgs5* ablation (Fig. 3E, F), indicating that the expression of these genes was controlled by RGS5*.* qPCR and immunoblotting assays confirmed the observations that RGS5 is crucial for the modulation of the immune response-associated signaling pathways in adult astrocytes (Fig. 3G–I).

### RGS5 enhances astrocytic pro-inflammatory response through interaction with TNFR1 or TNFR2

**Fig. 4:**
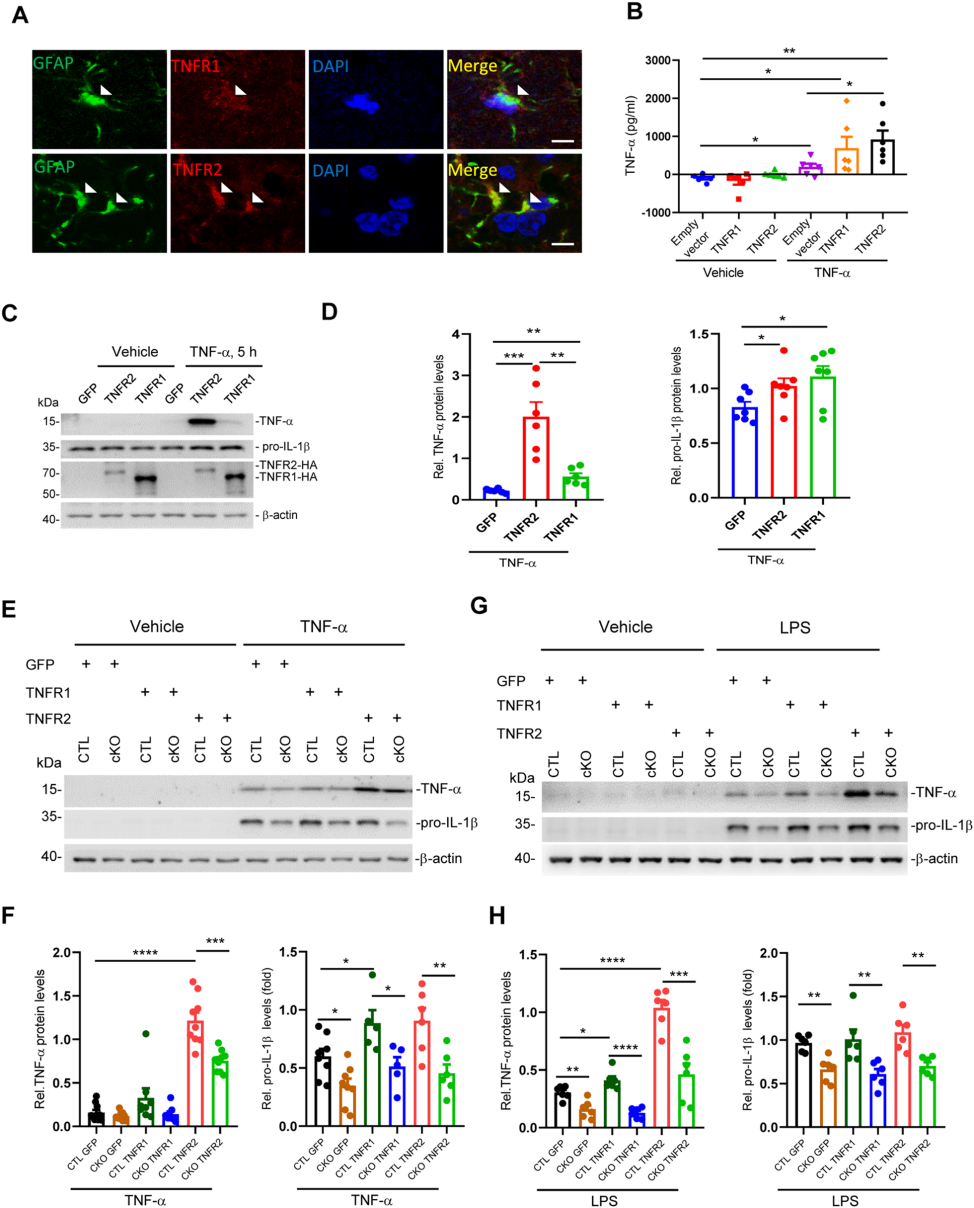
RGS5 is necessary for astrocytic TNFRs-promoted inflammatory factor expression with TNF-α/LPS challenge. **A** Double immunofluorescence staining for GFAP and TNFR1 or TNFR2 on the substantia nigra (SN). Arrows indicate the double-labeled cells. Scale bars, 5 μm. **B** ELISA analysis showing increased secretion of TNF-α in primary cultured astrocytes overexpressing TNF receptors mediated by lentivirus following exposure to TNF-α. The cells were incubated with TNF-α (100 ng/ml) for 1 h, and then washed 3 times with DMEM. Then, the culture medium was replaced with fresh DMEM containing 10% FBS and collected for ELISA 4 h later (*n* = 6). **C**, **D** Representative Western blots (**C**) and quantifications (**D**) showing endogenous TNF-α and pro-IL-1β protein expression in primary cultured astrocytes overexpressing GFP, TNFR1 or TNFR2 mediated by lentivirus transfection following exposure to TNF-α or vehicle for 5 h. The cells were washed three times with PBS before lysed. TNF-α 100 ng/ml, 5 h, *n* = 7. **E**–**H** Representative Western blots showing the reduction of TNF-α and pro-IL-1β protein levels in *Rgs5-null* (*RGS5*^*hGFAP*^) astrocytes. Primary astrocytes were transfected with Lentivirus (LV-GFP, LV-TNFR1 or LV-TNFR2) followed by challenge with either TNF-α (100 ng/ml) (**E**, **F**) or LPS (500 ng/ml) (**G, H**). *n* = 3–5. Data are expressed as mean ± SEM, two tailed *t*-test. *P < 0.05; **P < 0.01; ***P < 0.001; ****P < 0.0001

Next, we sought to investigate the mechanisms by which RGS5 modulates the immune response-associated signaling pathways. Given that canonical function of RGS5 is a regulator of receptors, such as GPCR [20, 50], and is found to be highly associated with TNF signaling pathway (Fig. 3), we speculated that RGS5 may be functionally related to TNFR signaling. Immunohistochemistry confirmed the expression of TNFRs in adult mouse astrocytes (Fig. 4A). Previous studies have shown that astrocytic TNFR2 has a neuroprotective activity, as its activation limits inflammatory response in animal models of PD or multiple sclerosis [51–55]. Unexpectedly, we found that LV-mediated overexpression of TNFR2 in primary cultured astrocytes elevated the secretion of pro-inflammatory mediator TNF-α following TNF-α challenge, as assessed by ELISA (Fig. 4B). Consistent with this, elevated expression of TNF-α and pro-IL1β in the cell lysates of these cells was also observed in primary cultured astrocytes overexpressing either TNFR1 or TNFR2 as compared to control (Fig. 4C, D). Interestingly, the promoting effects of TNFR1 and TNFR2 on inflammatory response was abrogated by *Rgs5*-deficiency (Fig. 4E, F), in accordance with the observations shown in *Rgs5* cKO mice (Fig. 1). *Rgs5*-deficient cells also showed the similar response following exposure to LPS (Fig. 4G, H), thus, independently confirming the validity of these findings.

**Fig. 5:**
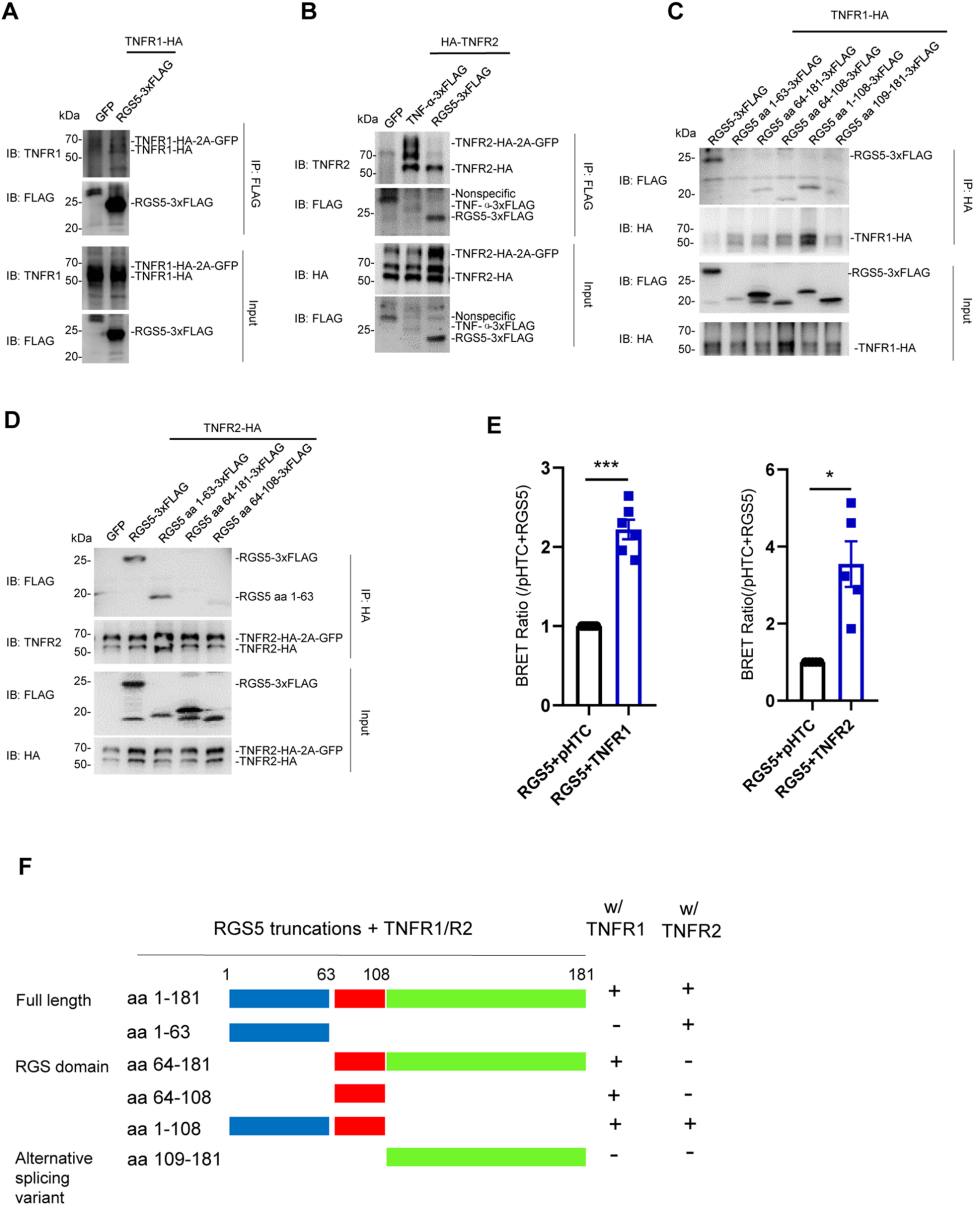
RGS5 interacts with either TNFR1 or TNFR2, respectively. **A**, **B** Co-immunoprecipitation (co-IP) assay reveals that RGS5 interacts with TNFR1 and TNFR2. HEK293T cells were transiently transfected with HA-tagged TNFR1 or TNFR2 and empty vector (GFP), mouse TNF-α-3xFLAG or RGS5-3xFLAG. **C**, **D** Identification of the RGS5 truncations that interact with TNFR1 (**C**) or TNFR2 (**D**). **E** NanoBRET protein:protein interaction assay reveals stronger interactions between RGS5 and TNFR1 or TNFR2 in transiently transfected cells. Cells were co-transfected with RGS5-NanoLuc and HaloTag-TNFR1/TNFR2, and the BRET ratios were normalized with that of RGS5-NanoLuc and empty vector containing HaloTag (pHTC). Experiments are repeated 5–6 times. **F** Diagram showing the bindings between RGS5 truncation mutants and TNF receptors. The assay was repeated at least three times. IB: immunoblot; IP: immunoprecipitation. Data are expressed as mean ± SEM. Student’s *t*-test. *P < 0.05; ***P < 0.001

The data showing functional association between RGS5 and TNFR receptors suggest that these proteins may function together in a complex to modulate inflammatory response. Co-IP assays revealed that RGS5 bound to the full-length TNFR1 or TNFR2 (Fig. 5A, B, Additional file 1: Fig. S4A, B). Further deletion analysis identified a fragment of RGS5 that mediated the interaction between RGS5 and TNFRs. Among a series of deletion mutants of RGS5, the RGS domain aa 64–108 was the minimal fragment for direct interaction between RGS5 and TNFR1, whereas the N-terminal fragment aa 1–63 of RGS5 was essential for the binding between RGS5 and TNFR2. In contrast, alternative splicing variant aa 109–181 (hereafter referred to as C-RGS5) did not bind to either TNFR1 or TNFR2 (Fig. 5C, D, F). Moreover, co-IP assays verified that both the N-terminal fragment aa 1–108 of RGS5 (hereafter referred to as N-RGS5) and the intracellular domain (ICD) of TNFR2 were required for the binding between RGS5 and TNFRs (Additional file 1: Fig. S4C–E). The results of binding between RGS5 and TNFRs were further confirmed in HEK293T cells using NanoBRET protein:protein interaction assay (Fig. 5E).

### Disruption of the interaction between RGS5 and TNFR suppresses astrocytic TNF-α production

**Fig. 6:**
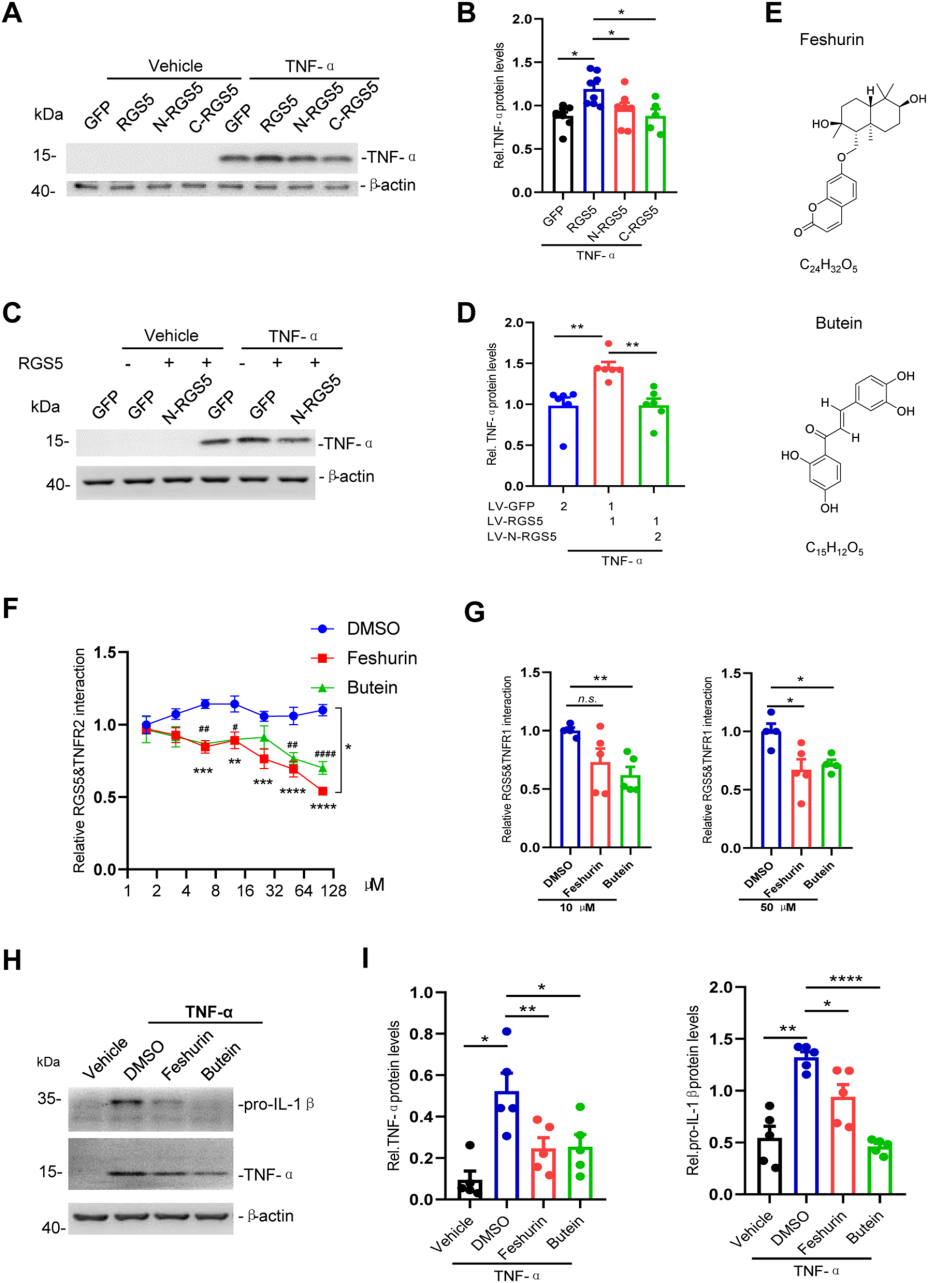
Interrupting RGS5–TNFR interaction suppresses astrocytic TNF-α production. **A** Representative Western blots showing TNF-α expression in primary cultured astrocytes transiently transfected with GFP, RGS5, N-RGS5 or C-RGS5. TNF-α (100 ng/ml) was added 48 h after transfection and incubated for 5 h. **B** Quantitative data shown in **A**. *n* = 5–8. **C** Representative Western blots showing a marked reduction of RGS5-mediated TNF-α expression by N-RGS5. Primary astrocytes were transfected with LV-GFP, LV-RGS5 or LV-N-RGS5. TNF-α (100 ng/ml) was added 72 h after transfection and incubated for 5 h. **D** Quantitative data shown in **C**. *n* = 5. **E** The structural formula of feshurin and butein. **F** NanoBit assay showing that treatment with feshurin or butein interrupts RGS5–TNFR2 interaction in a dose-dependent manner. Cells were incubated with feshurin or butein for 6 h at indicated concentrations. # indicates comparisons between vehicle (DMSO) and butein-treated groups; * indicates comparisons between vehicle (DMSO) and feshurin groups. (*n* = 4). **G** NanoBit assay showing that treatment with feshurin or butein interrupts RGS5–TNFR1 interaction at indicated concentrations. (*n* = 4–5). **H**, **I** Reduced pro-IL-1β and TNF-α expression in astrocytes pre-treated with feshurin or butein (50 µM) for 2 h and then challenged with TNF-α (20 ng/ml, 4 h) (*n* = 5). Data are expressed as mean ± SEM. Two-way ANOVA followed with Bonferroni’s multiple comparisons test or two-tailed *t-* test. *P < 0.05; **P < 0.01; ***P < 0.001; ****P < 0.0001

Next, we asked whether the physical RGS5–TNFR interaction is necessary for RGS5-mediated proinflammatory response. We assessed the contributions of the two major RGS5 fragments, N-RGS5 and C-RGS5 to the production of proinflammatory mediators. We found that neither of the two RGS5 fragments significantly altered TNF-α expression as compared to GFP control (Fig. 6A, B), and N-RGS5 was thus chosen to serve as a dominant negative form of RGS5. As expected, overexpression of N-RGS5 significantly reduced the elevation of TNF-α production induced by overexpression of full-length RGS5 (Fig. 6C, D) or co-expression of TNFR1 or TNFR2 in primary astrocytes following exposure to TNF-α (Additional file 1: Fig. S5A–D), indicating that the bindings between RGS5 and TNFR1 or TNFR2 is required for the TNFR signaling-induced pro-inflammatory mediator production.

Our data suggest that the proinflammatory effect of TNFR2 on astrocytes was more prominent compared to that of TNFR1 (Fig. 4B–D). This prompted us to investigate the underlying mechanisms of the RGS5–TNFR2 interaction. Previous studies have shown that the TNFR2 Thr377Ile mutation is associated with T cell lymphoma, including mycosis fungoides and Sézary syndrome. TNFR2 Thr377Ile expression led to increased TRAF2 degradation and enhanced NF-κB activation [56, 57]. Intriguingly, we found tyrosine phosphorylation of TNFR2 at T377 to be important for the interaction between RGS5 and TNFR2 during inflammatory response. Overexpression of TNFR2 T377I mutant resulted in a profound suppression of binding between TNFR2 and RGS5 leading to a marked reduction in the production of proinflammatory mediators as compared to wildtype TNFR2 in astrocytes exposed to TNF-α (Additional file 1: Fig. S5E–G). These data suggest that RGS5 regulates TNFR2-mediated immune response at least partially through modulation of TNFR2 phosphorylation.

Next, we sought to search for small molecular compounds which have an anti-inflammatory activity through interrupting RGS5–TNFR2 interaction. A natural compound library was screened by using RGS5–TNFR2 interaction-based NanoBit protein–protein assay. It was revealed that two compounds feshurin, also reported as samarcandin [58], and butein showed a profound inhibition on the RGS5–TNFR2 interaction in a dose-dependent manner (Fig. 6E, F). Likewise, feshurin and butein also interrupted RGS5–TNFR1 interaction in NanoBit protein–protein assay (Fig. 6G). Both compounds significantly suppressed NF-κB activity following TNF-α challenge, as assessed by NF-κB luciferase reporter activity assay (Additional file 1: Fig. S5H). Such inhibitory effects were also observed in primary cultured astrocytes, as pre-treatment with either feshurin or butein significantly reduced proinflammatory mediator expression induced by either TNF-α or pre-formed alpha-synuclein fibrils (PFFs) (Fig. 6H, I, Additional file 1: S5I, J). Altogether, these data suggest that blockade of the RGS5–TNFR interaction alleviates astrocytic proinflammatory response.

### RGS5 expression is transcriptionally controlled by EBF1

**Fig. 7:**
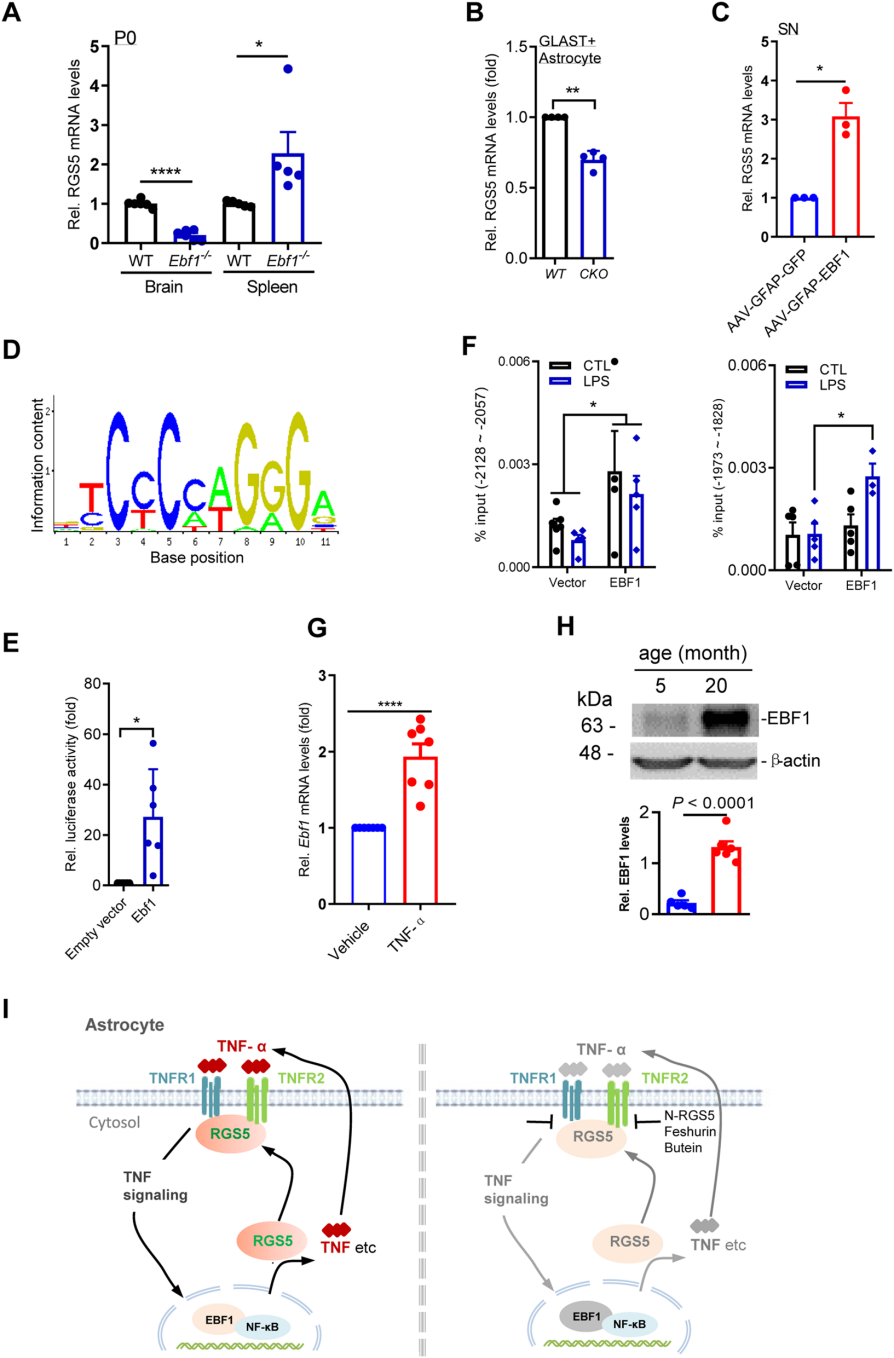
RGS5 expression is transcriptionally controlled by EBF1. **A** qPCR analysis shows RGS5 mRNA levels in the brain and spleen of *Ebf1* knockout mouse pups and their control at postnatal day 0. *n* = 5–6, Unpaired *t*-test. **B** Representative graph showing a reduction of RGS5 mRNA levels in GLAST^+^ astrocytes isolated from the whole brain of 2-month-old *Ebf1*^*hGFAP−CreER*^ mice. *n* = 5. **C** Representative graph showing *RGS5* mRNA levels in mice receiving stereotaxic intra-nigra injections of AAV-GFAP-EBF1 in ipsilateral side and AAV-GFAP-GFP in the contralateral side. The SN were harvested 3 weeks post-injection. *n* = 3, Paired *t*-test. **D** Prediction of EBF1 binding motif by using JASPAR online database. **E** Dual luciferase assay reveals that EBF1 binds to *RGS5* promoter. *RGS5* promoter was cloned into pGL3-Basic-luciferase reporter and was co-transfected into HEK293-T cells with an expression vector encoding *EBF1* cDNA (EF1α-EBF1) or empty vector (EF1α). Cells were harvested for dual luciferase assay 36 h after transfection. Values were normalized to those of empty vectors. *n* = 6, Paired *t-*test. **F** ChIP assay shows that multiple predicted EBF1 binding sequences in *RGS5* promoter regions were verified in HEK293T cells stably overexpressing EBF1 tagged with HA or control challenged with LPS (1 µg/ml, 2 h). *n* = 3–5, two-way ANOVA followed with Bonferroni’s multiple comparisons test. **G** Representative graph showing EBF1 mRNA expression in primary cultured astrocytes exposed to TNF-α (100 ng/ml) or vehicle for 5 h, respectively. *n* = 7, Paired *t-*test. **H** Immunoblotting for EBF1 in the SN of adult mice aged 5 or 20 months, *n* = 6. **I** Schematic drawing shows the EBF1/RGS5/TNFR-associated molecular machinery in astrocytes responding to immune stimuli TNF-α (left panel), and suppressed astrocytic inflammatory response by interrupting the interaction between RGS5 and TNFRs (right panel). Data are expressed as mean ± SEM. *P < 0.05; **P < 0.01; ****P < 0.0001

To understand the mechanisms that regulate RGS5-mediated astrogliosis, we sought to identify specific upstream mediators of RGS5 that may also play important roles in the regulation of the inflammatory response. Our previous study showed that transcription factor early B cell factor-1 (EBF1), a key determinant of early developmental processes in the B cell lineage [59–61] is also required for the development of mesencephalon [62]. We thus tested whether EBF1 controls RGS5 expression in the brain. It was revealed that *Ebf1* deficiency in the postnatal brain resulted in the loss of Rgs5 mRNA expression, suggesting that Rgs5 mRNA expression was highly dependent on EBF1 (Fig. 7A). Conversely, in the spleen, the largest peripheral immune organ, deficiency of *Ebf1* caused an overt increase in *Rgs5* mRNA expression (Fig. 7A). This antithetical data indicates distinct regulatory mechanisms of RGS5 expression in the CNS and peripheral immune system.

To investigate the astrocyte-specific Rgs5 regulation by EBF1, we generated *Ebf1*^*hGFAP−CreER*^ cKO mice from which GLAST^+^ astrocytes were isolated using MACS. We found that *Ebf1-null* astrocytes exhibited a reduction in *Rgs5* expression (Fig. 7B). Consistent with this result, AAV-mediated EBF1 overexpression driven by GFAP promotor in the SN augmented the expression levels of *Rgs5* mRNA (Fig. 7C), confirming that the regulatory role of EBF1 for RGS5 expression in astrocytes.

Next, we determined whether EBF1 directly regulates the transcription of *Rgs5*. Several EBF1 binding sites in regulatory regions upstream of *Rgs5* were predicted based on the JASPAR CORE database (Fig. 7D). A dual luciferase reporter assay showed that the reporter gene expression levels in the constructs containing *Rgs5* promoter segments were dramatically increased in HEK293T cells stably overexpressing *Ebf1* as compared with negative controls (Fig. 7E). Moreover, chromatin immunoprecipitation followed by PCR amplification of *Rgs5* promoter segments also indicated that EBF1 directly bound to the *Rgs5* promoter at multiple sites, including − 2128 to − -2057 bp and − 1973 to − 1828 bp from the transcriptional start site. LPS significantly enhanced the recruitment of EBF1 to the latter binding site (Fig. 7F). It was reported that EBF1 is one of the predicted transcription factors that associate with NF-κB and the motifs of NF-κB co-occur with EBF1 ± 50 bp [63, 64]. These data suggest that EBF1, as an instrumental gene, at least in vitro, transcriptionally controls *Rgs5* expression and may promote neuroinflammation through collaboration with NF-κB signaling.

We found that like RGS5, EBF1 expression was responsive to external stimuli, as manifested by elevated EBF1 mRNA expression in astrocytes responding to TNF-α in primary cultures (Fig. 7G). Moreover, EBF1 expression was also associated with aging, as there were higher levels of EBF1 protein in the brain of aged mice (20 months old) as compared to young animals (5 months old) (Fig. 7H). These results suggest that during aging and aging-related neurodegenerative disorders, EBF1 and RGS5 function in a collaborative manner to promote neuroinflammation during aging and brain diseases.

## Discussion

A wealth of evidence indicates that astrogliosis is highly associated with neuropathologies. Traditionally, astrogliosis has been viewed as passive response to neuronal injury. Damaged neurons and microglia have been reported to induce astrogliosis [65]. However, the mechanisms that lead to astrogliosis are not completely understood. In this study, we show that RGS5–TNFR signaling axis is a driving force for astrocyte activation during neurodegeneration. RGS5–TNFR signaling contributes to persistent production of proinflammatory mediators resulting in the self-propagation of glial response. These data demonstrate a crucial role of RGS5–TNFR axis in astrocyte-driven inflammation, providing new insights into the cellular and molecular mechanisms underlying chronic neuroinflammation in the pathogenesis of neurodegeneration, especially in regard to PD. This notion is consistent with our recent study that inactivation of astrocytic dopamine D2 receptor (Drd2)-6-pyruvoyl-tetrahydropterin synthase (PTS) axis is able to diminish astrogliosis and neurodegeneration in a mouse model of multiple sclerosis [66]. These studies together demonstrate a vital role of astrogliosis in influencing disease outcomes in neurodegenerative diseases.

It is known that RGS5 has anti-inflammation effects in peripheral tissue [67, 68], such as adipocytes [69, 70]. In contrast, we found here that RGS5 in the brain had opposite functions from those in the peripheral system in the modulation of inflammatory response. In the CNS, mice lacking *Rgs5* selectively in astrocytes displayed a marked attenuation of LPS-induced increases in pro-inflammatory mediator levels; whereas, in the peripheral tissues/cells, such as vascular endothelial cells, RGS5 has a distinct function, as shown by the loss of *Rgs5* exacerbating disease severity in an animal model of atherosclerosis partially through increased inflammatory mediator expression in vessels [67, 68]. Moreover, RGS5 transcription is distinctively regulated by EBF1 between the CNS and peripheral tissues (Fig. 7A), suggesting a unique property of astrocytic EBF1/RGS5 axis. Therefore, RGS5 is multifaceted protein in the complex biological processes and its regulatory roles may be dependent on its location and the context, suggesting distinct regulatory pathways used in the regulation of innate immunity between the CNS and peripheral systems.

One of the most interesting findings of the present study is that RGS5 switched the role of TNFR2 from neuroprotective to pro-inflammatory, leading to the increased production of cytokines. Previous studies have indicated that activation of TNFR signaling through the respective receptors leads to distinct outcomes. TNFR1 predominantly augments pro-inflammatory response, demyelination and neuronal death, whereas TNFR2 restrains pro-inflammatory milieu and promotes the initiation of tissue regeneration and remyelination in animal models of ischemia or multiple sclerosis [51–55]. Unexpectedly, in the present study we found that activation of astrocytic TNFR2 resulted in a remarkable pro-inflammatory response which is opposite to that previously thought. The diametric regulatory mechanism and outcome for TNFR2 signaling suggest that blockade of both TNFR1and TNFR2 signaling in astrocytes is required for the suppression of CNS inflammation, representing a new therapeutic strategy for CNS inflammation which is distinct from those proposed for autoimmune disease [54].

We found that RGS5 enhanced TNFR signaling via either TNFR1 or TNFR2, utilizing a unique regulatory mechanism in astrocytes. Canonical role of RGS proteins is a suppressor for a variety of GPCR signaling through binding with Gα subunits [20–22]. In contrast, our findings indicate that astrocytic RGS5 functions in a Gα-independent manner, as RGS5 binds to TNFR1 or TNFR2, a single transmembrane receptor, causing an enhanced production of pro-inflammatory mediators. Similar to TNF-α, the expression of IL-1β decreases in astrocytes with RGS5 ablation. However, there is no evidence that IL-1β is induced by RGS5 directly, or it is one of the downstream effectors of RGS5 contributing to inflammatory response as shown in Fig. 3C–F. Our work raised a probability that RGS5 enhances IL1 signaling via interacting with IL1 receptor which should be further investigated. Thus, our findings indicate that, beyond its traditional role in GPCR signaling, astrocytic RGS5 serves as a switch of TNF signaling circuit with resultant activation of astrocytes thereby contributing to chronic neuroinflammation and neurodegeneration. Our data expand the scope of receptors that RGS proteins can regulate, and its association with brain diseases.

Moreover, RGS5-mediated activation of TNFR signaling can be precluded by using small molecular compounds feshurin and butein. Feshurin is one of the sesquiterpene coumarins from *Ferula samarkandica* Korovin, which protects against testicular ischemia/reperfusion injury and controls the inflammatory mediators [58, 71]. Relatively, butein, a biologically active flavonoid that is known to be ubiquitous in medicinal plants, shows anti-inflammatory effects in multiple types of cells [72–75]. Our findings here provide new insights into the molecular mechanism underlying the anti-inflammatory effects of the two compounds.

Furthermore, RGS5-mediated activation of TNFR signaling can be disrupted by overexpression of the N-terminal fragment aa 1–108 of RGS5, serving as a dominant negative form of RGS5 for competing with the function of full-length RGS5. The findings presented here provide unique advantages over targeting the upstream TNFRs. TNFRs are wildly expressed in a variety of tissues/cells. Their expression can be regulated by multiple mechanisms. To the best of our knowledge, astrocyte-specific regulatory machinery for TNFR signaling has not been identified. The finding that co-expression of RGS5 and TNFR in astrocytes would provide an increased level of specificity over targeting the TNFR for regulating astrocyte activities.

It is conceivable that with the unique EBF1-controlled RGS5/TNFR protein machinery, astrocytes serve not only as a signal transducer for TNF-α signals, but also, more importantly, as signal modulators during inflammatory process. Indeed, as shown in Fig. 7I, extracellular immune signals control the expression of EBF1 which in turn influences astrocytic expression of pro-inflammatory mediator via RGS5, forming an autocrine signal loop that contributes to the excessive production of cytokines and persistent neuroinflammation during neurodegeneration. It has been believed that during neuroinflammation, microglia expressing neurotoxic proteins drive astrocyte-mediated inflammatory response and subsequent innate immune cell-mediated neuronal cell death, with the resultant misfolded protein and cell debris contributing to additional inflammation [76]. Thus, the astrocyte-based autocrine signal loop revealed in the present study likely collaborate with the existing vicious cycle involving the microglia–astrocyte–neuron, causing stronger inflammatory response that may last even longer.

In summary, our findings reveal a previously unchartered role for RGS5 in the modulation of the TNFR-mediated inflammatory response in astrocytes. The identification of the RGS5/TNFR signaling provides an opportunity to control neuroinflammation-associated machinery. The development of a therapeutic strategy, that blocks the interaction between RGS5 and TNFRs, may effectively limit chronic neuroinflammation, thereby interfering with the progression of neurodegenerative diseases.

### Supplementary Information

**Additional file 1: Figure S1.** Selective ablation of *Rgs5* in astrocytes inhibits LPS-induced inflammation in vivo. (**A**) Double immunofluorescence staining for GFAP and RGS5 on the SN. Arrowheads indicate the double-labeled cells. Scale bar, 10 μm. (**B**) Double immunofluorescence staining for GFAP and RGS5 on the spinal cord of A30P-mutant α-synuclein transgenic mice or littermate control. (**C**) Quantification of immunofluorescence integrated density for RGS5 shown in **B**. Scale bar, 10 μm. (**D**) Representative graph showing a reduction in Rgs5 mRNA expression in the SN and STR of *Rgs5*^*hGFAP*^ cKO mice. *n* = 3–9. (**E**) Immunohistochemical staining of TH on the ventral mesencephalon of adult *Rgs5*^*mGFAP*^ cKO and their littermate controls administered with a single intra-nigral injection of LPS. Scale bar, 100 μm. (**F**) Quantitative data of TH^+^ cell numbers shown in **E** (*n* = 8). (**G**) Representative graph showing a reduction in Rgs5 mRNA expression in the SN or GLAST^+^ astrocytes of *Rgs5*^*hGFAP−CreER*^ cKO mice (2 months old). *Rgs5*^*hGFAP−CreER*^ cKO mice were administered with tamoxifen (i.p.) and killed 3-week post-injection. *n* = 4–5. (**H**) Representative graph showing a reduction in Rgs5 mRNA expression in *Rgs5*^*mGFAP*^ cKO astrocytes (*RGS5*-null). *n* = 5. Data are expressed as mean ± SEM. *P < 0.05; **P < 0.01; ***P < 0.001; ****P < 0.001. **Figure S2.** Selective ablation of *Rgs5* in astrocytes inhibits 6-OHDA-induced DA neuron lose and glial activation in the SN. (**A**) Immunohistochemical staining for TH, GFAP and IBA1 on the striatum or ventral mesencephalon of adult *Rgs5*^*hGFAP−CreER*^ cKO and their littermate controls received a single striatal injection of 6-OHDA (4 µg). Scale bars, 100 μm. (**B**) Quantification of TH positive cells shown in **A**, *n* = 5. (**C**, **D**) Quantification of immunoreactive cells or immunoreactivity for GFAP (**C**) or IBA1 (**D**) shown in **A**. *n* = 5. Data are expressed as mean ± SEM, two-tailed *t-* test. *P < 0.05. **Figure S3.** Increased production of pro-inflammatory mediators induced by *Rgs5* overexpression in vitro*.* (**A**) Representative fluorescent microphotographs of rat primary cultured astrocytes transfected with either Lenti-RGS5-HA-FLAG-2A-GFP (LV-RGS5) driven by CMV promoter or Lenti-control vector (LV-GFP) for 12 h and followed by treatment with LPS or vehicle. Cells were harvested 48 h later. Scale bars, 100 μm. (**B**) Representative graphs showing relative mRNA levels of indicated pro-inflammatory mediators in the transfected astrocytes with or without LPS challenge (500 ng/ml, 5 h). *n* = 4–8 per group. (**C**) NF-κB luciferase reporter assay in HEK293T cells co-expressing the NF-κB-Luc reporter and RGS5 or GFP shows the NF-κB luciferase reporter activities in cells incubated with TNF-α (20 ng/ml). The assay was performed in triplicates for 3 times. Data are expressed as mean ± SEM. Two-way ANOVA. *P < 0.05; ****P < 0.001. **Figure S4.** RGS5 interacts with TNFR1, TNFR2 or TNFR2-ICD, respectively. (**A**, **B**) Co-immunoprecipitation assay reveals that RGS5 interacts with TNFR1 and TNFR2. HEK293T cells were transiently transfected with HA-tagged TNFR1 or TNFR2 and empty vector (GFP), mouse TNF-α-3xFLAG or RGS5-3xFLAG. The assay is repeated at least three times. IB, immunoblot; IP, immunoprecipitation. (**C**-**E**) Co-immunoprecipitation assays show the interaction between RGS5 truncation mutants and the intracellular domain (ICD) of TNFR2. C2A-RGS5 refers to the cysteine-2-alanine mutant of RGS5 which slows down the degradation of the protein. RGS5 aa 1–108, but not RGS5 aa 109–181, interacts with TNFR2-ICD. **Figure S5.** Interrupting RGS5–TNFR interaction suppresses astrocytic TNF-α production. (**A**-**D**) Representative Western blots showing N-RGS5-induced attenuation of increased TNF-α expression in primary astrocytes following co-overexpression of RGS5 with TNFR1 (**A**, **B**) or TNFR2 (**C**, **D**). Primary astrocytes were transfected with lentivirus (∼10^6^ infectious units per ml) followed by challenge with TNF-α (100 ng/ml). (*n* = 5). (**E**) Identification of RGS5 fragments that interact with TNFR2 or TNFR2(T377I). Experiments are repeated 4 times. (**F**) Reduced TNF-α expression in astrocytes transfected with TNFR2(T377I) compared to WT TNFR2 exposed to LPS. (**G**) Quantification of data shown in **F** (*n* = 5). (**H**) NF-κB luciferase reporter activities in HEK293T cells. Cells were pre-incubated with feshurin or butein for 2 h at indicated concentrations and challenged with TNF-α (20 ng/ml, 4 h). # indicates comparisons between DMSO and butein groups. * indicates comparisons between DMSO and feshurin groups.  (**I**, **J**) Reduced pro-IL-1β expression in astrocytes pre-incubated with feshurin or butein (50 µM) for 2 h and then challenged with PFFs (2 µg/ml, 4 h) (*n* = 4). Data are expressed as mean ± SEM. Two-way ANOVA followed with Bonferroni’s multiple comparisons test or two-tailed *t-* test. *P < 0.05; **P < 0.01; ***P < 0.001; ****P < 0.0001.

## Data Availability

The datasets supporting the conclusions of this article are available from the corresponding author upon reasonable request.
