# Supplementary material for: RGS5 augments astrocyte activation and facilitates neuroinflammation via TNF signaling
Source: J Neuroinflammation. 2023 Sep 6;20:203. doi: 10.1186/s12974-023-02884-w (PMC10481574; doi:10.1186/s12974-023-02884-w)
Supplement: Supplementary file 1 — Additional file 1: Figure S1. Selective ablation of Rgs5 in astrocytes inhibits LPS-induced inflammation in vivo. (A) Double immunofluorescence staining for GFAP and RGS5 on the SN. Arrowheads indicate the double-labeled cells. Scale bar, 10 μm. (B) Double immunofluorescence staining for GFAP and RGS5 on the spinal cord of A30P-mutant α-synuclein transgenic mice or littermate control. (C) Quantification of immunofluorescence integrated density for RGS5 shown in B. Scale bar, 10 μm. (D) Representative graph showing a reduction in Rgs5 mRNA expression in the SN and STR of Rgs5hGFAP cKO mice. n = 3–9. (E) Immunohistochemical staining of TH on the ventral mesencephalon of adult Rgs5mGFAP cKO and their littermate controls administered with a single intra-nigral injection of LPS. Scale bar, 100 μm. (F) Quantitative data of TH+ cell numbers shown in E (n = 8). (G) Representative graph showing a reduction in Rgs5 mRNA expression in the SN or GLAST+ astrocytes of Rgs5hGFAP−CreER cKO mice (2 months old). Rgs5hGFAP−CreER cKO mice were administered with tamoxifen (i.p.) and killed 3-week post-injection. n = 4–5. (H) Representative graph showing a reduction in Rgs5 mRNA expression in Rgs5mGFAP cKO astrocytes (RGS5-null). n = 5. Data are expressed as mean ± SEM. *P < 0.05; **P < 0.01; ***P < 0.001; ****P < 0.001. Figure S2. Selective ablation of Rgs5 in astrocytes inhibits 6-OHDA-induced DA neuron lose and glial activation in the SN. (A) Immunohistochemical staining for TH, GFAP and IBA1 on the striatum or ventral mesencephalon of adult Rgs5hGFAP−CreER cKO and their littermate controls received a single striatal injection of 6-OHDA (4 µg). Scale bars, 100 μm. (B) Quantification of TH positive cells shown in A, n = 5. (C, D) Quantification of immunoreactive cells or immunoreactivity for GFAP (C) or IBA1 (D) shown in A. n = 5. Data are expressed as mean ± SEM, two-tailed t- test. *P < 0.05. Figure S3. Increased production of pro-inflammatory mediators induced by Rgs5 overexpression [file 12974_2023_2884_MOESM1_ESM.pdf]

Figure S1

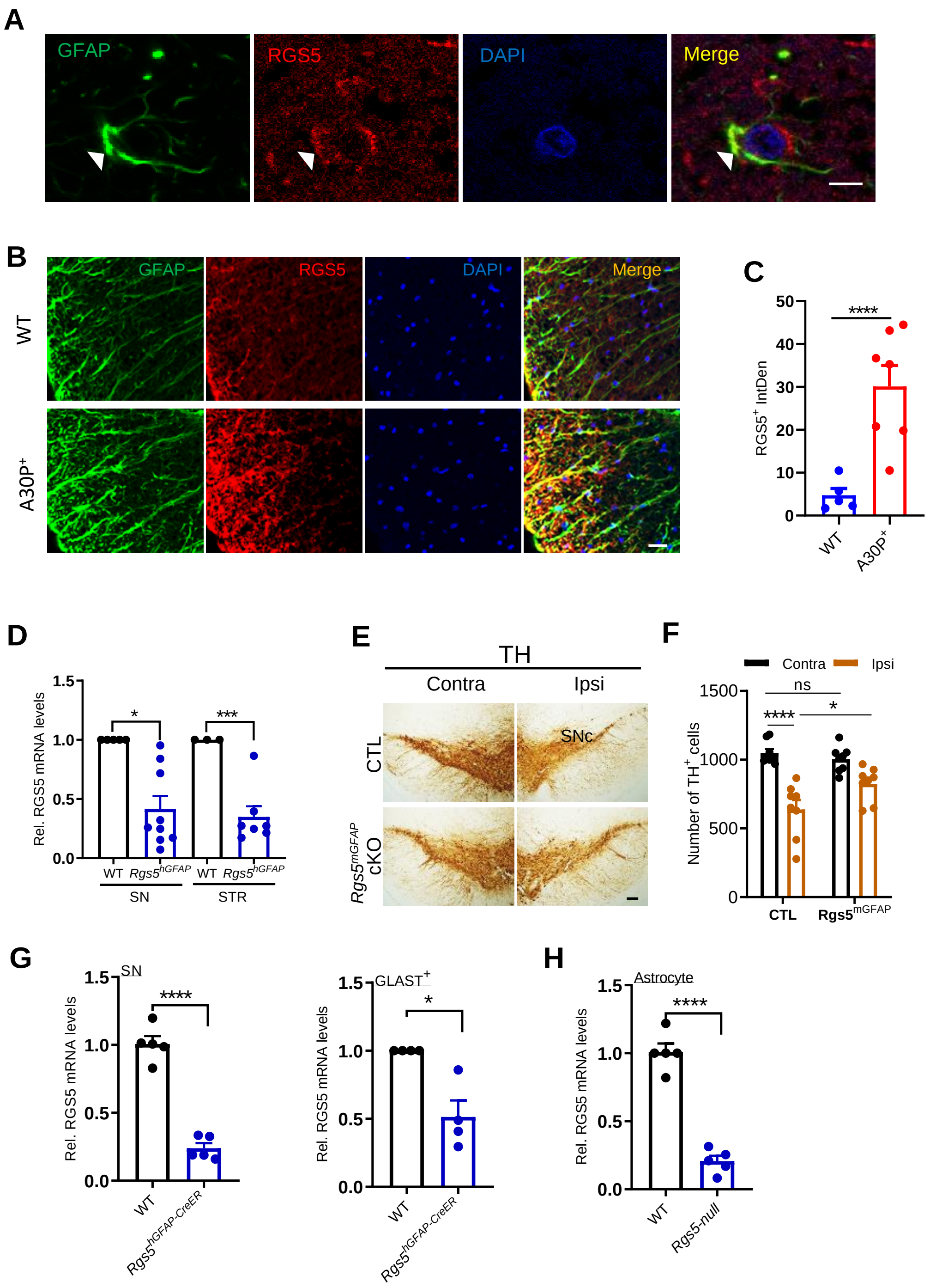

A
GFAP
RGS5
DAPI
Merge
B
WT
A30P+
GFAP
RGS5
DAPI
Merge
C
RGS5+ IntDen
****
50
40
30
20
10
0
WT
A30P+
D
Rel. RGS5 mRNA levels
1.5
1.0
0.5
0.0
*
***
WT Rgs5hGFAP WT Rgs5hGFAP
SN
STR
E
TH
Contra
Ipsi
CTL
SNc
Rgs5mGFAP cKO
F
Contra Ipsi
ns
****
*
Number of TH+ cells
1500
1000
500
0
CTL
Rgs5mGFAP
G
SN
****
Rel. RGS5 mRNA levels
WT
Rgs5hGFAP-CreER
GLAST+
*
H
Astrocyte
****
WT
Rgs5-null

Figure S1. Selective ablation of *Rgs5* in astrocytes inhibits LPS-induced inflammation *in vivo*.

(A) Double immunofluorescence staining for GFAP and RGS5 on the SN. Arrowheads indicate the double-labeled cells. Scale bar, 10 μm.

(B) Double immunofluorescence staining for GFAP and RGS5 on the spinal cord of A30P-mutant α-synuclein transgenic mice or littermate control.

(C) Quantification of immunofluorescence integrated density for RGS5 shown in (B). Scale bar, 10 μm.

(D) Representative graph showing a reduction in Rgs5 mRNA expression in the SN and STR of $Rgs5^{hGFAP}$ cKO mice. *n* = 3 - 9.

(E) Immunohistochemical staining of TH on the ventral mesencephalon of adult $Rgs5^{mGFAP}$ cKO and their littermate controls administered with a single intra-nigral injection of LPS. Scale bar, 100 μm.

(F) Quantitative data of TH^+ cell numbers shown in (E) (*n* = 8).

(G) Representative graph showing a reduction in Rgs5 mRNA expression in the SN or $GLAST^+$ astrocytes of $Rgs5^{hGFAP\text{-}CreER}$ cKO mice (2-month-old). $Rgs5^{hGFAP\text{-}CreER}$ cKO mice were administered with tamoxifen (i.p.) and sacrificed 3 week post injection. *n* = 4-5.

(H) Representative graph showing a reduction in Rgs5 mRNA expression in $Rgs5^{mGFAP}$ cKO astrocytes (*RGS5*-null). *n* = 5.

Data are expressed as mean ± SEM. *$P < 0.05$; **$P < 0.01$; ***$P < 0.001$; ****$P < 0.0001$.

Figure S2

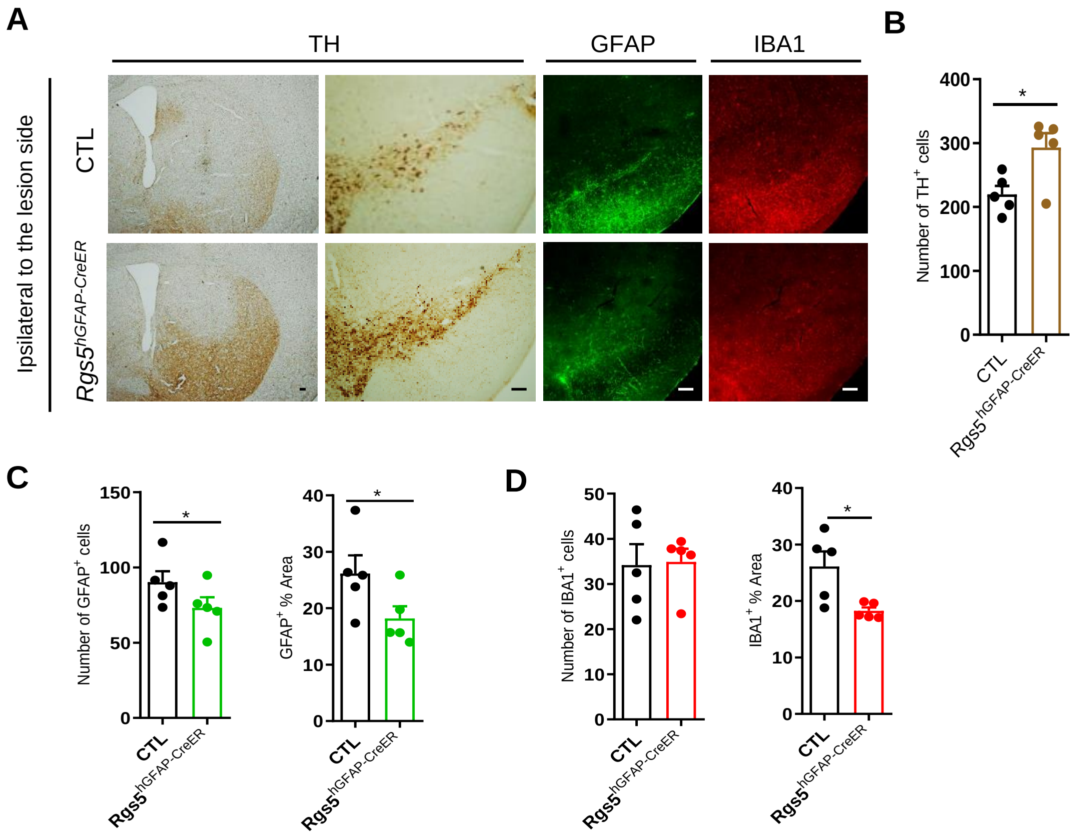

Figure S2. Selective ablation of *Rgs5* in astrocytes inhibits 6-OHDA-induced DA neuron lose and glial activation in the SN.

(A) Immunohistochemical staining for TH, GFAP and IBA1 on the striatum or ventral mesencephalon of adult *Rgs5*$^{hGFAP\text{-}CreER}$ cKO and their littermate controls received a single striatal injection of 6-OHDA (4 μg). Scale bars, 100 μm.

(B) Quantification of TH positive cells shown in (A), n = 5.

(C, D) Quantification of immunoreactive cells or immunoreactivity for GFAP (C) or IBA1 (D) shown in (A). n = 5. Data are expressed as mean ± SEM, two-tailed t- test. $^*P < 0.05$.

Figure S3

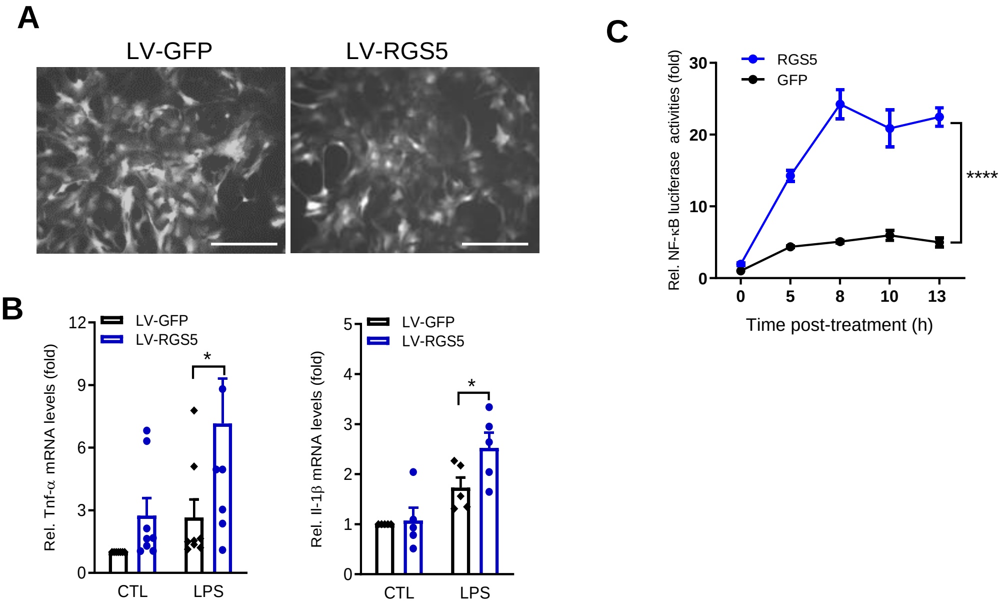

Figure S3. Increased production of pro-inflammatory mediators induced by *Rgs5* overexpression *in vitro*.

(A) Representative fluorescent microphotographs of rat primary cultured astrocytes transfected with either Lenti-RGS5-HA-FLAG-2A-GFP (LV-RGS5) driven by CMV promoter or Lenti-control vector (LV-GFP) for 12 h and followed by treatment with LPS or vehicle. Cells were harvested 48 h later. Scale bars, 100 µm.

(B) Representative graphs showing relative mRNA levels of indicated pro-inflammatory mediators in the transfected astrocytes with or without LPS challenge (500 ng/ml, 5 h). $n = 4 - 8$ per group.

(C) NF-κB luciferase reporter assay in HEK293T cells co-expressing the NF-κB-Luc reporter and RGS5 or GFP shows the NF-κB luciferase reporter activities in cells incubated with TNF-α (20 ng/ml). The assay was performed in triplicates for 3 times. Data are expressed as mean ± SEM. Two-way ANOVA. $*P < 0.05$; $****P < 0.0001$.

Figure S4

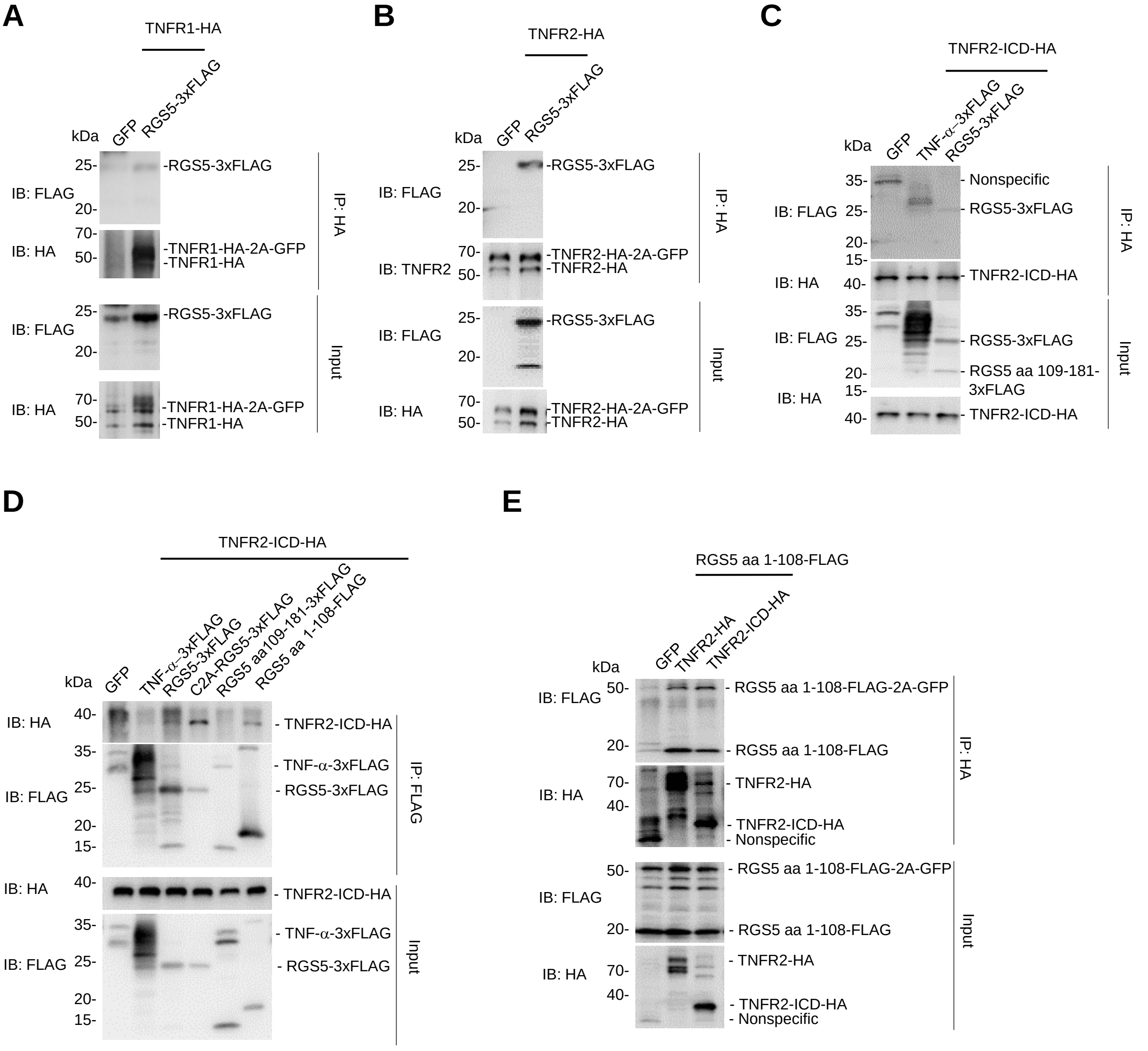

Figure S4. RGS5 interacts with TNFR1, TNFR2 or TNFR2-ICD, respectively.

(A, B) Co-immunoprecipitation assay reveals that RGS5 interacts with TNFR1 and TNFR2. HEK293T cells were transiently transfected with HA-tagged TNFR1 or TNFR2 and empty vector (GFP), mouse TNF-α-3xFLAG or RGS5-3xFLAG. The assay was repeated at least three times. IB, immunoblot; IP, immunoprecipitation.

(C-E) Co-immunoprecipitation assays show the interaction between RGS5 truncation mutants and the intracellular domain (ICD) of TNFR2. C2A-RGS5 refers to the cysteine-2-alanine mutant of RGS5 which slows down the degradation of the protein. RGS5 aa 1-108, but not RGS5 aa 109-181, interacts with TNFR2-ICD.

Figure S5

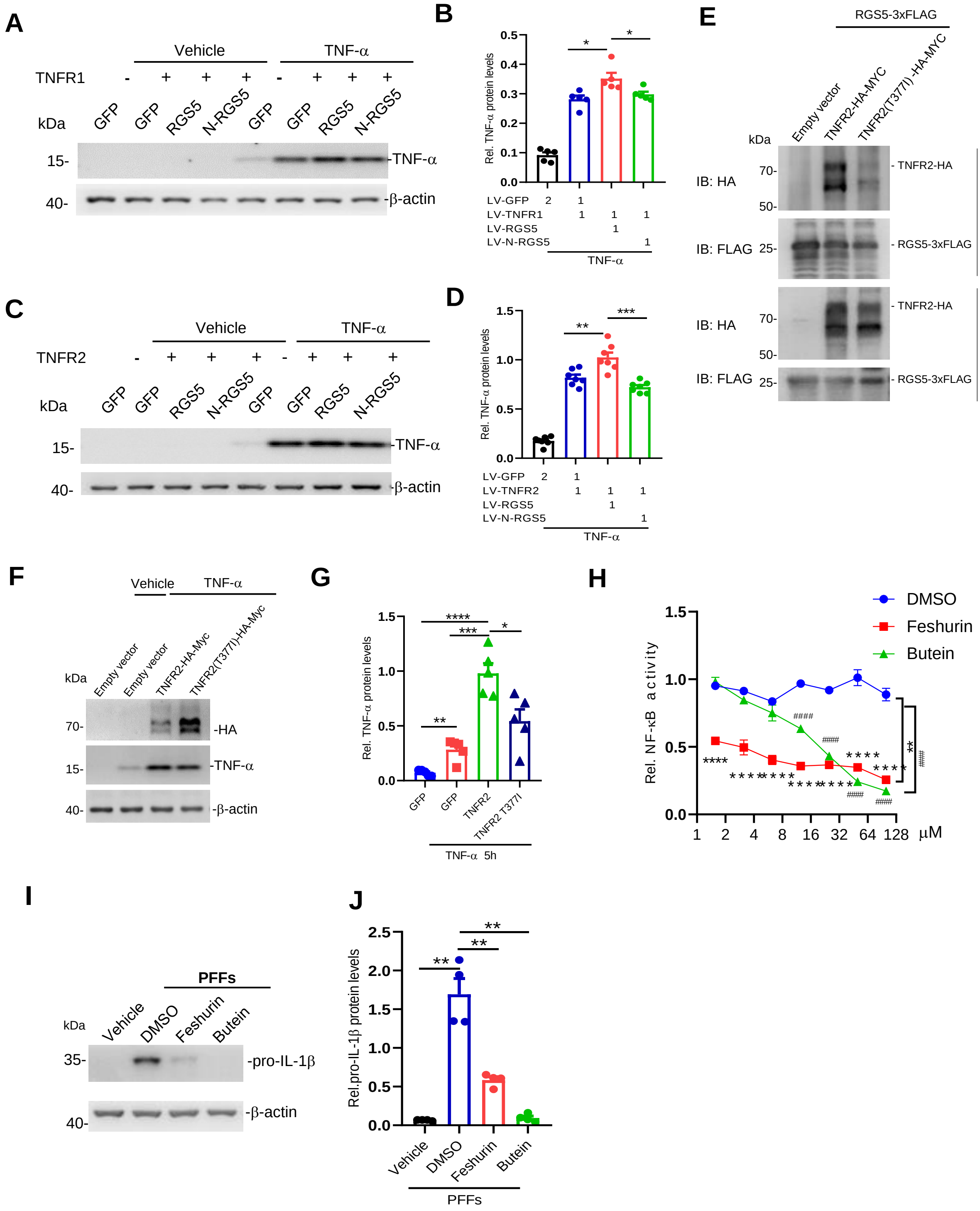

A
Vehicle
TNF-α
TNFR1
GFP
RGS5
N-RGS5
kDa
15-
40-
-TNF-α
-β-actin
B
Rel. TNF-α protein levels
LV-GFP
LV-TNFR1
LV-RGS5
LV-N-RGS5
TNF-α
C
TNFR2
D
LV-TNFR2
E
RGS5-3xFLAG
Empty vector
TNFR2-HA-MYC
TNFR2(T377I) -HA-MYC
IB: HA
IB: FLAG
TNFR2-HA
RGS5-3xFLAG
IP: FLAG
Input
F
TNFR2-HA-Myc
TNFR2(T377I)-HA-Myc
-HA
G
TNFR2
TNFR2 T377I
TNF-α 5h
H
DMSO
Feshurin
Butein
Rel. NF-κB activity
μM
I
PFFs
Vehicle
-pro-IL-1β
J
Rel.pro-IL-1β protein levels

Figure S5. Interrupting RGS5-TNFR interaction suppresses astrocytic TNF-α production.

(A-D) Representative Western blots showing N-RGS5-induced attenuation of increased TNF-α expression in primary astrocytes following co-overexpression of RGS5 with TNFR1 (A and B) or TNFR2 (C and D). Primary astrocytes were transfected with lentivirus ($\sim 10^6$ infectious units per ml) followed by challenge with TNF-α (100 ng/ml). (n = 5).

(E) Identification of RGS5 fragments that interact with TNFR2 or TNFR2(T377I). Experiments are repeated 4 times.

(F) Reduced TNF-α expression in astrocytes transfected with TNFR2(T377I) compared to WT TNFR2 exposed to LPS.

(G) Quantification of data shown in (F) (n = 5).

(H) NF-κB luciferase reporter activities in HEK293T cells. Cells were pre-incubated with feshurin or butein for 2 h at indicated concentrations and challenged with TNF-α (20 ng/ml, 4h). # indicates comparisons between DMSO and Butein groups; * indicates comparisons between DMSO and feshurin groups.

(I, J) Reduced pro-IL-1β expression in astrocytes pre-incubated with feshurin or butein (50 μM) for 2 h and then challenged with PFFs (2 μg/ml, 4 h) (n = 4).

Data are expressed as mean ± SEM, two-way ANOVA followed with Bonferroni's multiple comparisons test or two-tailed t- test. *$P < 0.05$; **$P < 0.01$; ***$P < 0.001$; ****$P < 0.0001$.
